# Supplementary material for: MERTK mediated novel site Akt phosphorylation alleviates SAV1 suppression
Source: Nat Commun. 2019 Apr 3;10:1515. doi: 10.1038/s41467-019-09233-7 (PMC6447540; doi:10.1038/s41467-019-09233-7)
Supplement: Supplementary file 1 — Supplementary Information [file 41467_2019_9233_MOESM1_ESM.pdf]

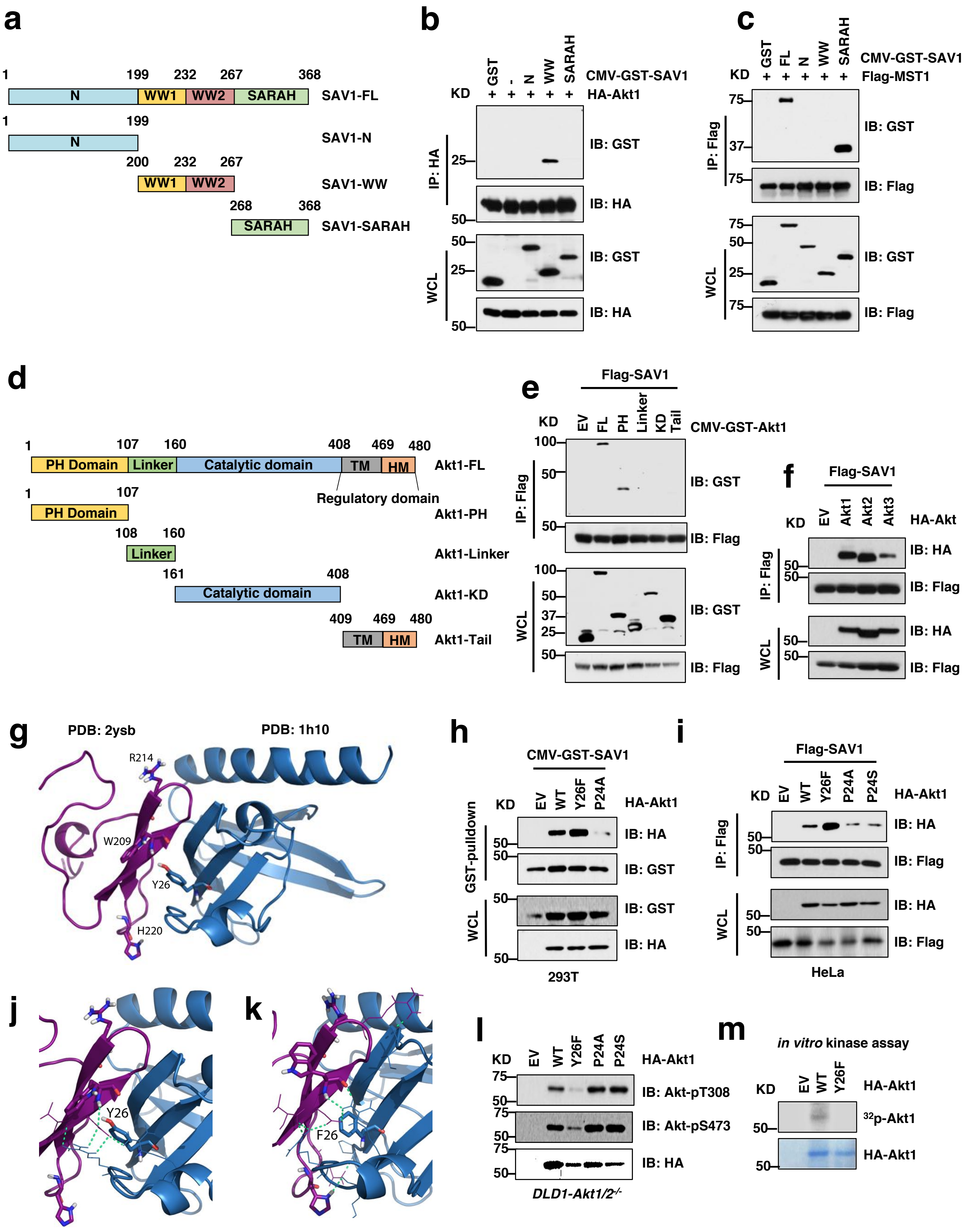
a
SAV1-FL
SAV1-N
SAV1-WW
SAV1-SARAH
N
WW1
WW2
SARAH
b
CMV-GST-SAV1
HA-Akt1
GST
-
N
WW
SARAH
KD
IP: HA
WCL
IB: GST
IB: HA
c
CMV-GST-SAV1
Flag-MST1
FL
IP: Flag
IB: Flag
d
PH Domain
Linker
Catalytic domain
TM
HM
Regulatory domain
Akt1-FL
Akt1-PH
Akt1-Linker
Akt1-KD
Akt1-Tail
e
Flag-SAV1
EV
PH
KD
Tail
CMV-GST-Akt1
f
HA-Akt
Akt1
Akt2
Akt3
g
PDB: 2ysb
PDB: 1h10
R214
W209
Y26
H220
h
GST-pulldown
WT
Y26F
P24A
293T
i
P24S
HeLa
j
k
F26
l
IB: Akt-pT308
IB: Akt-pS473
DLD1-Akt1/2-/-
m
in vitro kinase assay
32p-Akt1
HA-Akt1

Supplementary Figure 1. Akt binds the WW domain of SAV1 through a PxY motif in its PH domain. (a) A cartoon illustration of domain structure for SAV1. (b-c) Immunoblot (IB) analyses of HA (b) or Flag (c) immunoprecipitates (IP) and whole cell lysates (WCL) derived from HEK293 cells transfected with indicated DNA constructs. (d) A cartoon illustration of domain structure for Akt1. (e-f) IB analyses of Flag-IP and WCL derived from HEK293 cells transfected with indicated DNA constructs. (g, j and k). A simulation model for the SAV1-WW domain/Akt1-PH domain structure. Please refer to the method section for details. (h) IB analysis of GST-pulldowns and WCL derived from HEK293T cells transfected with indicated DNA constructs. (i) IB analysis of Flag-IP and WCL derived from HeLa cells transfected with indicated DNA constructs. (l) IB analysis of *DLD1-Akt1/2*$^{-/-}$ cells transfected with indicated DNA constructs. (m) *In vitro* kinase assays to examine Akt activity. Please refer to the method section for details.

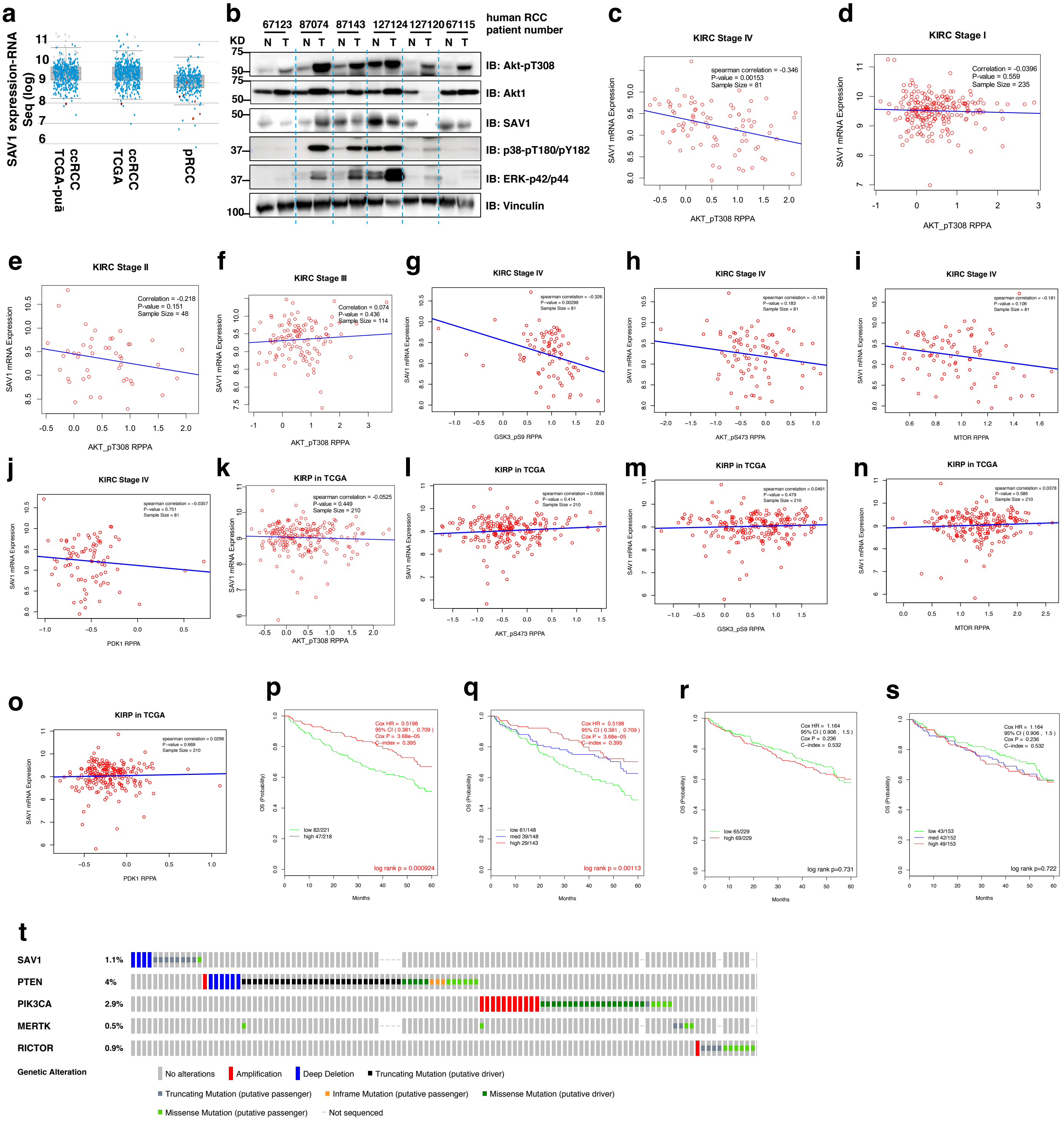

a
SAV1 expression-RNA Seq (log)
ccRCC TCGA-puā
ccRCC TCGA
pRCC
b
human RCC patient number
67123 87074 87143 127124 127120 67115
N T N T N T N T N T N T
KD
IB: Akt-pT308
IB: Akt1
IB: SAV1
IB: p38-pT180/pY182
IB: ERK-p42/p44
IB: Vinculin
c
KIRC Stage IV
spearman correlation = -0.346
P-value = 0.00153
Sample Size = 81
SAV1 mRNA Expression
AKT_pT308 RPPA
d
KIRC Stage I
Correlation = -0.0396
P-value = 0.559
Sample Size = 235
SAV1 mRNA Expression
AKT_pT308 RPPA
e
KIRC Stage II
Correlation = -0.218
P-value = 0.151
Sample Size = 48
SAV1 mRNA Expression
AKT_pT308 RPPA
f
KIRC Stage III
Correlation = 0.074
P-value = 0.436
Sample Size = 114
SAV1 mRNA Expression
AKT_pT308 RPPA
g
KIRC Stage IV
spearman correlation = −0.326
P−value = 0.00299
Sample Size = 81
SAV1 mRNA Expression
GSK3_pS9 RPPA
h
KIRC Stage IV
spearman correlation = −0.149
P−value = 0.183
Sample Size = 81
SAV1 mRNA Expression
AKT_pS473 RPPA
i
KIRC Stage IV
spearman correlation = −0.181
P−value = 0.106
Sample Size = 81
SAV1 mRNA Expression
MTOR RPPA
j
KIRC Stage IV
spearman correlation = −0.0357
P−value = 0.751
Sample Size = 81
SAV1 mRNA Expression
PDK1 RPPA
k
KIRP in TCGA
spearman correlation = -0.0525
P-value = 0.449
Sample Size = 210
SAV1 mRNA Expression
AKT_pT308 RPPA
l
KIRP in TCGA
spearman correlation = 0.0566
P−value = 0.414
Sample Size = 210
SAV1 mRNA Expression
AKT_pS473 RPPA
m
KIRP in TCGA
spearman correlation = 0.0491
P−value = 0.479
Sample Size = 210
SAV1 mRNA Expression
GSK3_pS9 RPPA
n
KIRP in TCGA
spearman correlation = 0.0378
P−value = 0.586
Sample Size = 210
SAV1 mRNA Expression
MTOR RPPA
o
KIRP in TCGA
spearman correlation = 0.0296
P−value = 0.669
Sample Size = 210
SAV1 mRNA Expression
PDK1 RPPA
p
Cox HR = 0.5198
95% CI (0.381 , 0.709)
Cox P = 3.68e−05
C−index = 0.395
low 82/221
high 47/218
log rank p = 0.000924
OS (Probability)
Months
q
Cox HR = 0.5198
95% CI (0.381 , 0.709)
Cox P = 3.68e−05
C−index = 0.395
low 61/148
med 39/148
high 29/143
log rank p = 0.00113
OS (Probability)
Months
r
Cox HR = 1.164
95% CI (0.906 , 1.5)
Cox P = 0.236
C−index = 0.532
low 65/229
high 69/229
log rank p=0.731
OS (Probability)
Months
s
Cox HR = 1.164
95% CI (0.906 , 1.5)
Cox P = 0.236
C−index = 0.532
low 43/153
med 42/152
high 49/153
log rank p=0.722
OS (Probability)
Months
t
SAV1 1.1%
PTEN 4%
PIK3CA 2.9%
MERTK 0.5%
RICTOR 0.9%
Genetic Alteration
No alterations
Amplification
Deep Deletion
Truncating Mutation (putative driver)
Truncating Mutation (putative passenger)
Inframe Mutation (putative passenger)
Missense Mutation (putative driver)
Missense Mutation (putative passenger)
Not sequenced

Supplementary Figure 2. An inverse correlation between SAV1 expression and Akt activation is observed in RCC patients. (a) mRNA expression data obtained from RNA-seq experiments in TCGA provided by cBioportal. (b) Immunoblot (IB) analysis of whole cell lysates (WCL) derived from RCC patient samples. N: non-tumor, T: tumor. (c-f) Examination of SAV1 expression (from TCGA KIRC RNA-seq data) and Akt-pT308 levels (from TCGA KIRC RPPA data) in kidney cancer patients with indicated disease stage. (g-j) Examination of the correlation of SAV1 expression (from TCGA KIRC RNA-seq data) with GSK3-pS9 (g), Akt-pS473 (h), mTOR (i) and PDK1 (j) protein levels (from TCGA KIRC RPPA data) in stage IV kidney cancer patients. (k) Examination of the correlation of SAV1 expression (from TCGA KIRP RNA-seq data) with Akt-pT308 (k), Akt-pS473 (l), GSK3-pS9 (m), mTOR (n) and PDK1 (o) protein levels (from TCGA KIRP RPPA data) in stage IV kidney cancer patients. (p-s) Kaplan-Meier plots indicating either low SAV1 expression or high Akt activity predicts poor prognosis. p and q: green line (low SAV1 expression); blue line (medium SAV1 expression) and red line (high SAV1 expression). r and s: red line (high Akt-pT308); blue line (medium Akt-pT308) and green line (low Akt-pT308). (t) Oncoprint from cBioportal indicating that SAV1 genetic changes are not concurrent with genetic alternations with canonical Akt upstream signaling components such as *PTEN* and *PIK3CA*.

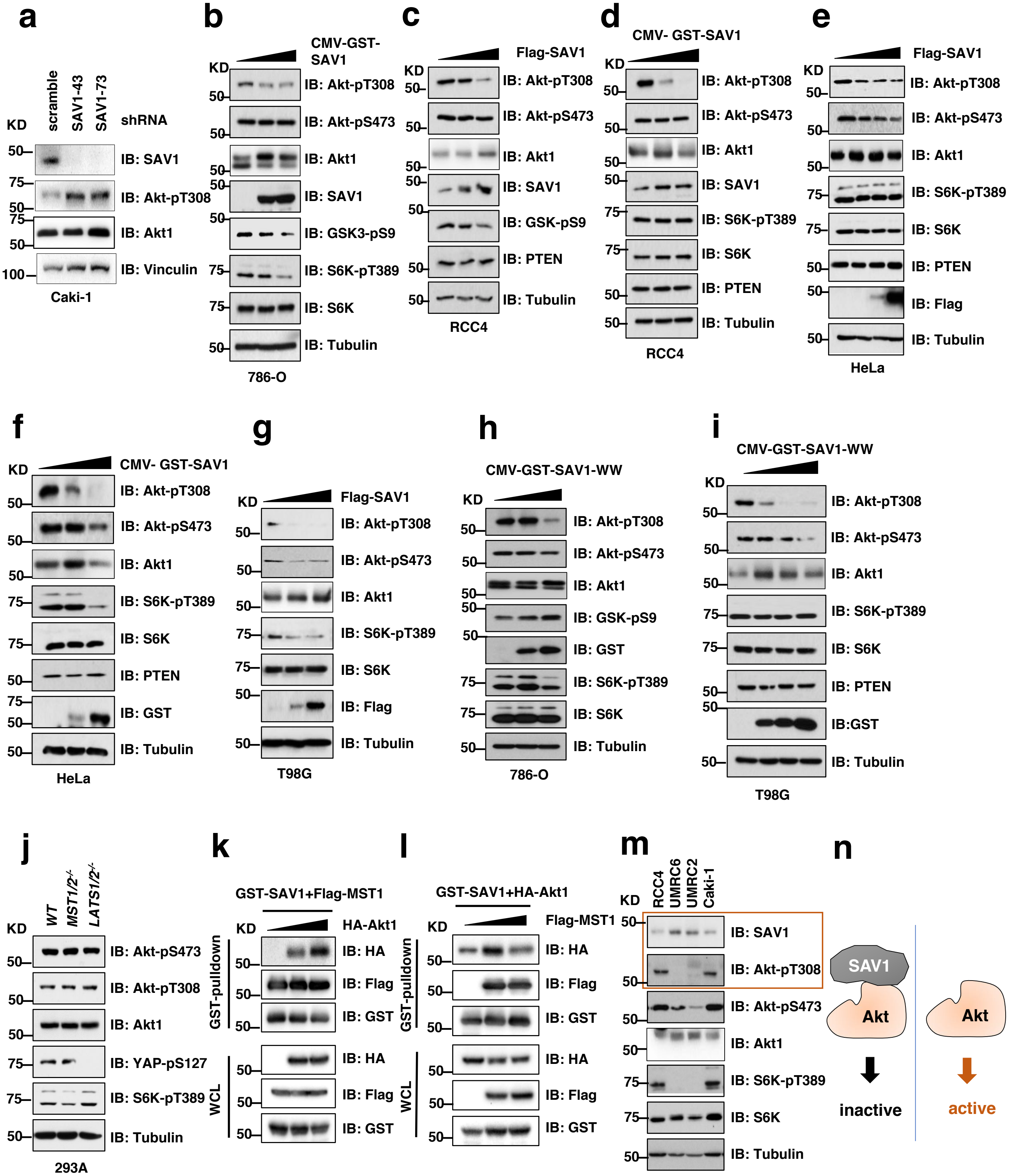
a
scramble
SAV1-43
SAV1-73
shRNA
KD
IB: SAV1
IB: Akt-pT308
IB: Akt1
IB: Vinculin
Caki-1
b
CMV-GST-SAV1
IB: Akt-pT308
IB: Akt-pS473
IB: Akt1
IB: SAV1
IB: GSK3-pS9
IB: S6K-pT389
IB: S6K
IB: Tubulin
786-O
c
Flag-SAV1
IB: Akt-pT308
IB: Akt-pS473
IB: Akt1
IB: SAV1
IB: GSK-pS9
IB: PTEN
IB: Tubulin
RCC4
d
CMV- GST-SAV1
IB: Akt-pT308
IB: Akt-pS473
IB: Akt1
IB: SAV1
IB: S6K-pT389
IB: S6K
IB: PTEN
IB: Tubulin
RCC4
e
Flag-SAV1
IB: Akt-pT308
IB: Akt-pS473
IB: Akt1
IB: S6K-pT389
IB: S6K
IB: PTEN
IB: Flag
IB: Tubulin
HeLa
f
CMV- GST-SAV1
IB: Akt-pT308
IB: Akt-pS473
IB: Akt1
IB: S6K-pT389
IB: S6K
IB: PTEN
IB: GST
IB: Tubulin
HeLa
g
Flag-SAV1
IB: Akt-pT308
IB: Akt-pS473
IB: Akt1
IB: S6K-pT389
IB: S6K
IB: Flag
IB: Tubulin
T98G
h
CMV-GST-SAV1-WW
IB: Akt-pT308
IB: Akt-pS473
IB: Akt1
IB: GSK-pS9
IB: GST
IB: S6K-pT389
IB: S6K
IB: Tubulin
786-O
i
CMV-GST-SAV1-WW
IB: Akt-pT308
IB: Akt-pS473
IB: Akt1
IB: S6K-pT389
IB: S6K
IB: PTEN
IB:GST
IB: Tubulin
T98G
j
WT
MST1/2-/-
LATS1/2-/-
IB: Akt-pS473
IB: Akt-pT308
IB: Akt1
IB: YAP-pS127
IB: S6K-pT389
IB: Tubulin
293A
k
GST-SAV1+Flag-MST1
HA-Akt1
GST-pulldown
WCL
IB: HA
IB: Flag
IB: GST
l
GST-SAV1+HA-Akt1
Flag-MST1
GST-pulldown
WCL
IB: HA
IB: Flag
IB: GST
m
RCC4
UMRC6
UMRC2
Caki-1
IB: SAV1
IB: Akt-pT308
IB: Akt-pS473
IB: Akt1
IB: S6K-pT389
IB: S6K
IB: Tubulin
n
SAV1
Akt
inactive
Akt
active

Supplementary Figure 3. The WW domain of SAV1 binds Akt and suppresses Akt activity. (a) Immunoblot (IB) analysis of whole cell lysates (WCL) derived from Caki-1 cells depleted of endogenous SAV1 by shRNAs. (b-g) IB analysis of WCL derived from 786-O (b), RCC4 (c-d), HeLa (e-f) and T98G (g) cells transfected with increasing doses of indicated SAV1 constructs. (h-i) IB analysis of WCL derived from 786-O (h) or T98G (i) cells that ectopically express GST-tagged SAV1-WW domain constructs. (j) IB analysis of WCL derived from HEK293A cells deleted of indicated endogenous genes. (k-l) IB analysis of GST-pulldown and WCL derived from HEK293 cells transfected with indicated DNA constructs. (m) IB analysis of WCL derived from indicated RCC cells. (n) A cartoon illustration of SAV1-mediated Akt activity inhibition.

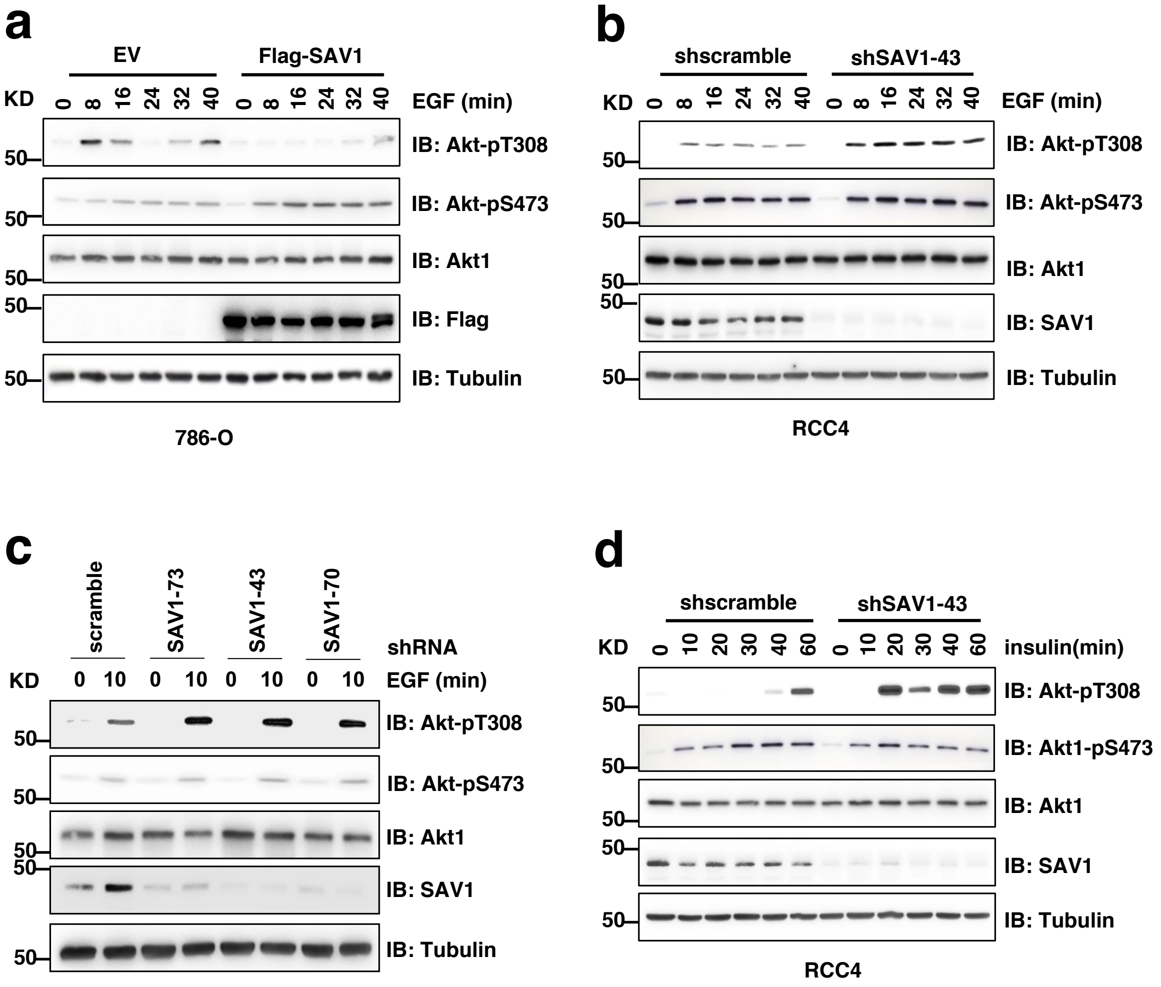
a
EV
Flag-SAV1
KD
0 8 16 24 32 40 0 8 16 24 32 40
EGF (min)
IB: Akt-pT308
IB: Akt-pS473
IB: Akt1
IB: Flag
IB: Tubulin
50
786-O
b
shscramble
shSAV1-43
KD
0 8 16 24 32 40 0 8 16 24 32 40
EGF (min)
IB: Akt-pT308
IB: Akt-pS473
IB: Akt1
IB: SAV1
IB: Tubulin
RCC4
c
scramble
SAV1-73
SAV1-43
SAV1-70
shRNA
KD
0 10 0 10 0 10 0 10
EGF (min)
IB: Akt-pT308
IB: Akt-pS473
IB: Akt1
IB: SAV1
IB: Tubulin
d
shscramble
shSAV1-43
KD
0 10 20 30 40 60 0 10 20 30 40 60
insulin(min)
IB: Akt-pT308
IB: Akt1-pS473
IB: Akt1
IB: SAV1
IB: Tubulin
RCC4

Supplementary Figure 4. SAV1 suppresses Akt-T308 phosphorylation upon EGF or insulin stimulation. (a) Immunoblot (IB) analysis of whole cell lysates (WCL) derived from 786-O cells transfected with indicated DNA constructs. Where indicated, cells were serum starved overnight and then treated with 100 nM EGF for indicated time periods before cell collection. (b-d) IB analyses of WCL derived from RCC4 (b, d) or Caki-1 (c) cells depleted of endogenous SAV1. Where indicated, cells were was serum starved overnight and then treated with 100 nM EGF (b, c) or insulin (d) for indicated time periods before cell collection.

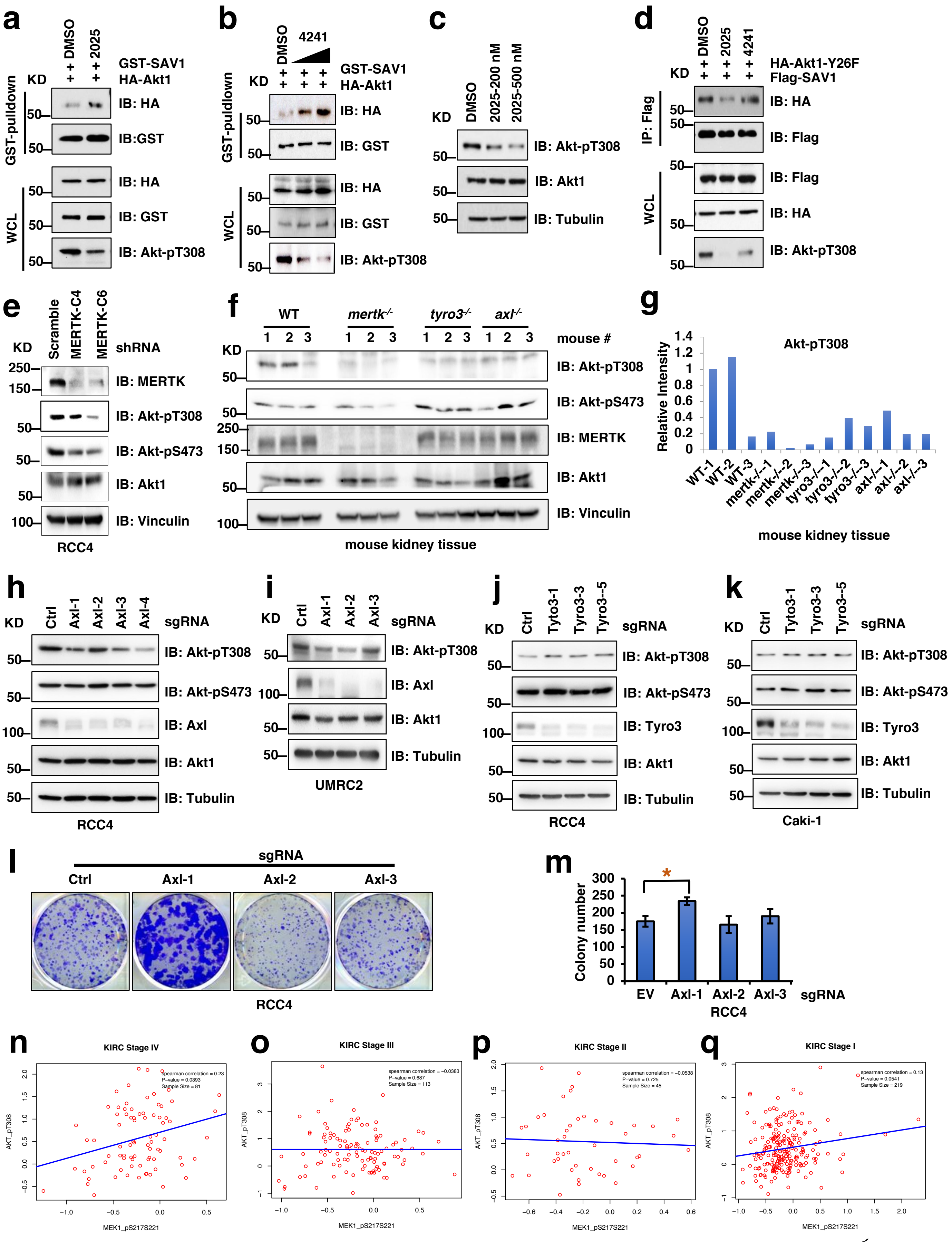
a
KD
+ DMSO
+ 2025
+ +
GST-SAV1
HA-Akt1
GST-pulldown
WCL
IB: HA
IB:GST
IB: HA
IB: GST
IB: Akt-pT308
50
b
DMSO
4241
GST-SAV1
HA-Akt1
IB: HA
IB: GST
IB: Akt-pT308
c
DMSO
2025-200 nM
2025-500 nM
IB: Akt-pT308
IB: Akt1
IB: Tubulin
d
+ DMSO
+ 2025
+ 4241
HA-Akt1-Y26F
Flag-SAV1
IP: Flag
IB: Flag
e
Scramble
MERTK-C4
MERTK-C6
shRNA
250
150
100
IB: MERTK
IB: Akt-pS473
IB: Akt1
IB: Vinculin
RCC4
f
WT
mertk-/-
tyro3-/-
axl-/-
1 2 3
mouse #
IB: MERTK
mouse kidney tissue
g
Akt-pT308
Relative Intensity
WT-1
WT-2
WT-3
mertk-/--1
mertk-/--2
mertk-/--3
tyro3-/--1
tyro3-/--2
tyro3-/--3
axl-/--1
axl-/--2
axl-/--3
h
Ctrl
Axl-1
Axl-2
Axl-3
Axl-4
sgRNA
IB: Axl
i
UMRC2
j
Tyto3-1
Tyro3-3
Tyro3--5
IB: Tyro3
k
Caki-1
l
sgRNA
Ctrl
Axl-1
Axl-2
Axl-3
RCC4
m
Colony number
EV
*
n
KIRC Stage IV
spearman correlation = 0.23
P-value = 0.0393
Sample Size = 81
AKT_pT308
MEK1_pS217S221
o
KIRC Stage III
spearman correlation = -0.0383
P-value = 0.687
Sample Size = 113
p
KIRC Stage II
spearman correlation = -0.0538
P-value = 0.725
Sample Size = 45
q
KIRC Stage I
spearman correlation = 0.13
P-value = 0.0541
Sample Size = 219

Supplementary Figure 5. MERTK inhibition leads to enhanced Akt1/SAV1 binding and attenuated Akt activity. (a-b) Immunoblot (IB) analysis of GST-pulldowns and whole cell lysates (WCL) derived from HeLa cells transfected with indicated DNA constructs. Where indicated, 300 nM UNC2025 (a) or 300 nM UNC4241 (b) was added for 2 hrs before cells were harvested. (c) IB analysis of WCL derived from 293T cells treated with indicated doses of UNC2025 for 2 hrs. (d) IB analysis of Flag-IP and WCL derived from HEK293 cells transfected with indicated DNA constructs. Where indicated, 300 nM UNC2025 or 300 nM UNC4241 was added for 2 hrs before cells were harvested. (e) IB analysis of WCL derived from RCC4 cells depleted of endogenous MERTK by shRNAs. (f-g) IB analysis of WCL derived from kidney tissues obtained from mice with indicated genotypes (f) and corresponding quantification for Akt-pT308 signals (g). (h-i) IB analysis of WCL derived from RCC4 (h) or UMRC2 (i) cells depleted of endogenous Axl by shRNAs. Infected cells were selected with 1 μg/ml puromycin for 72 hours to eliminate non-infected cells before cell collection. (j-k) IB analysis of WCL derived from RCC4 (j) or Caki-1 (k) cells depleted of endogenous Tyro3 by shRNAs. Infected cells were selected with 1 μg/ml puromycin for 72 hours to eliminate non-infected cells before cell collection. (l-m) 600 cells obtained in (h) were plated on 6-well plates and 15 days later formed colonies were fixed and visualized by crystal violet staining (l) and quantified (m). (n-q) Examination of Akt-pT308 levels (from TCGA KIRC RPPA data) and MEK1-pS217pS221 levels (an indicator of MERTK activity, from TCGA KIRC RPPA data) in kidney cancer patients with indicated disease stage.

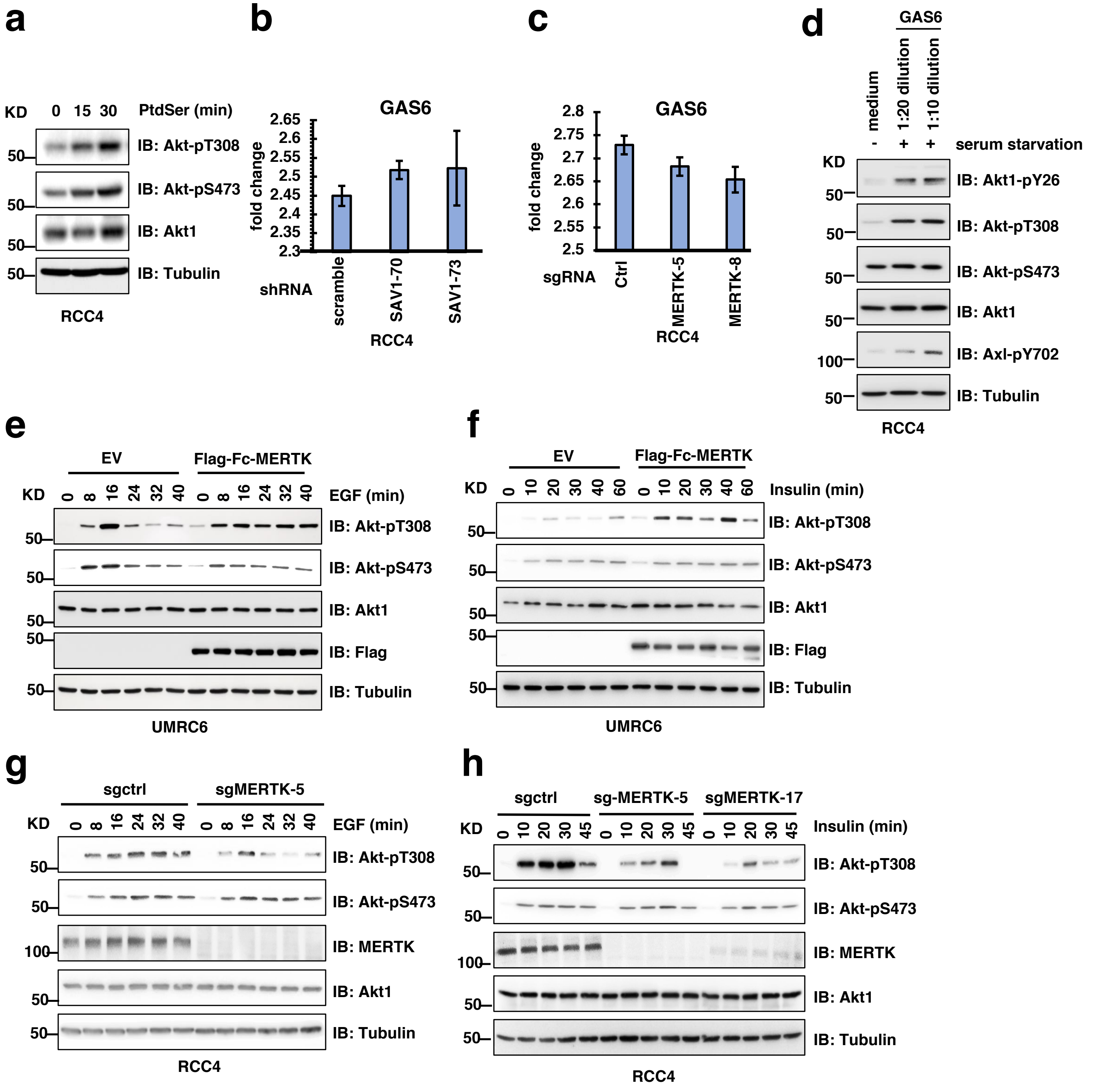
a
KD
0 15 30
PtdSer (min)
IB: Akt-pT308
IB: Akt-pS473
IB: Akt1
IB: Tubulin
RCC4
b
GAS6
fold change
shRNA
scramble
SAV1-70
SAV1-73
RCC4
c
GAS6
fold change
sgRNA
Ctrl
MERTK-5
MERTK-8
RCC4
d
GAS6
medium
1:20 dilution
1:10 dilution
serum starvation
IB: Akt1-pY26
IB: Akt-pT308
IB: Akt-pS473
IB: Akt1
IB: Axl-pY702
IB: Tubulin
RCC4
e
EV
Flag-Fc-MERTK
EGF (min)
IB: Akt-pT308
IB: Akt-pS473
IB: Akt1
IB: Flag
IB: Tubulin
UMRC6
f
EV
Flag-Fc-MERTK
Insulin (min)
IB: Akt-pT308
IB: Akt-pS473
IB: Akt1
IB: Flag
IB: Tubulin
UMRC6
g
sgctrl
sgMERTK-5
EGF (min)
IB: Akt-pT308
IB: Akt-pS473
IB: MERTK
IB: Akt1
IB: Tubulin
RCC4
h
sgctrl
sg-MERTK-5
sgMERTK-17
Insulin (min)
IB: Akt-pT308
IB: Akt-pS473
IB: MERTK
IB: Akt1
IB: Tubulin
RCC4

Supplementary Figure 6. METK activation promotes Akt-T308 phosphorylation. (a) IB analysis of WCL derived from RCC4 cells that were serum starved overnight and stimulated with 80 μM PtdSer for the indicated time period. (b-c) RT-PCR indicating that GAS6 are expressed in RCC4 cells. (d) IB analysis of WCL derived from RCC4 cells that were serum starved overnight and stimulated with indicated dilutions of home-made GAS6 (from Earp lab) for 30min before cell collection. (e-f) IB analysis of WCL derived from UMRC6 cells transfected with indicated constructs. Where indicated, cells were serum starved overnight and stimulated with either EGF (e) or insulin (f) for indicated time periods before cell collection. (g-h) IB analysis of WCL derived from indicated RCC4 cells. Where indicated, cells were serum starved overnight and stimulated with either EGF (g) or insulin (h) for indicated time periods before cell collection.

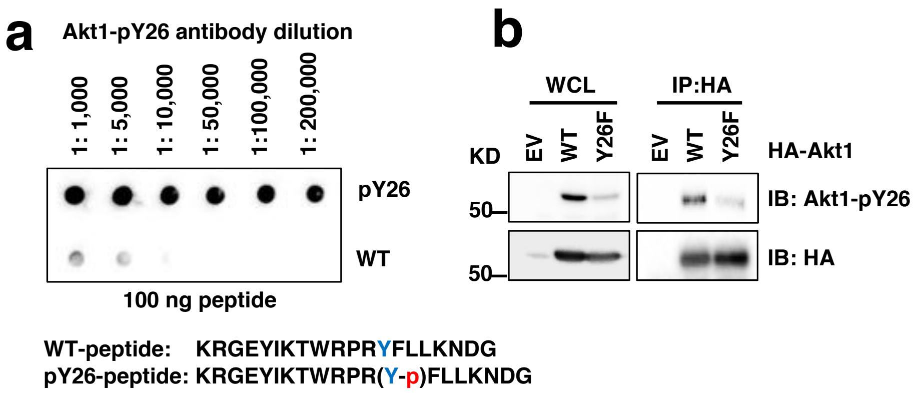

Supplementary Figure 7. Validation of the Akt1-pY26 antibody. (a) Dot blot assays to examine the specificity for the Akt1-pY26 antibody. 100 ng of the antigen peptides were spotted on nitrocellulose membrane and the Akt1-pY26 antibody was diluted with indicated factors and used for western blot analyses. (b) IB analyses of HA-IP and WCL derived from HEK293 cells transfected with indicated HA-Akt1 constructs.

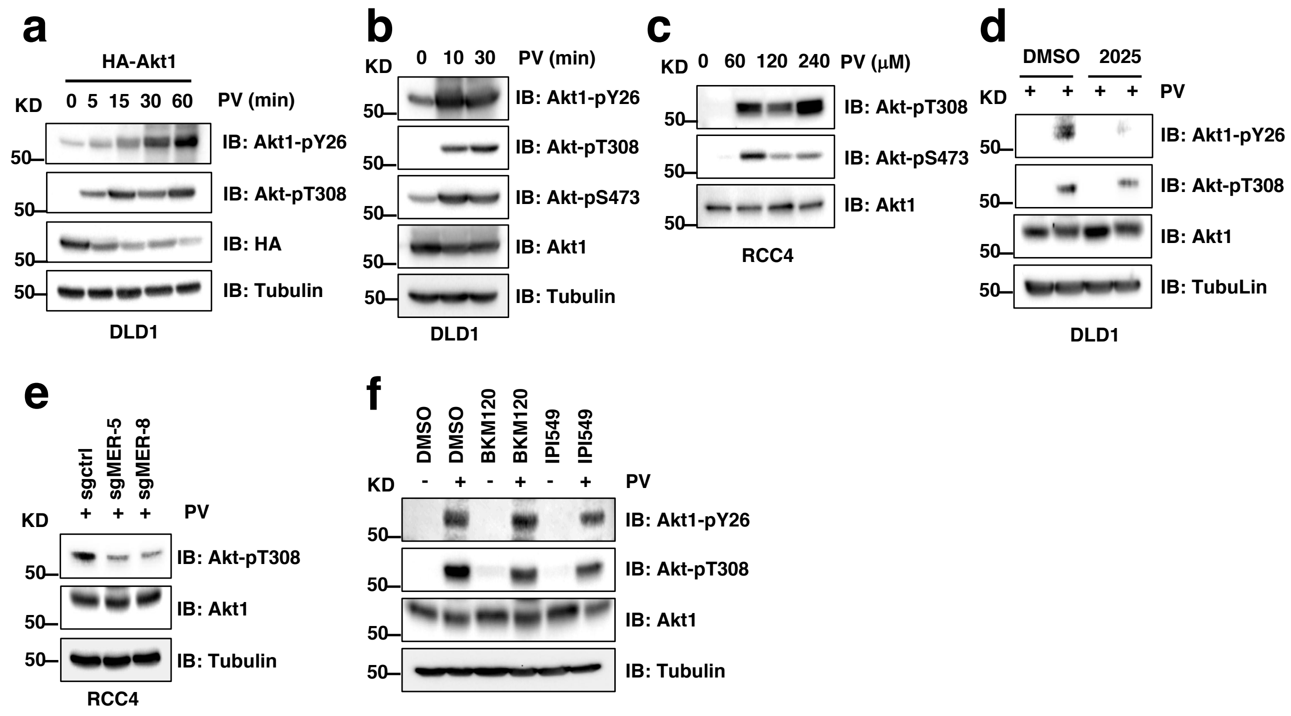

Supplementary Figure 8. MERTK activation facilitates Akt1-pY26 and Akt activation. (a) IB analysis of WCL derived from DLD1 cells transfected with HA-Akt1 and treated with 60 μM PV for indicated time periods. (b-c) IB analysis of WCL derived from DLD1 (b) or RCC4 (c) cells treated with 60 μM PV for indicated time periods. (d) IB analysis of WCL derived from DLD1 cells treated with 60 μM PV for 30min. Where indicated, 300 nM UNC2025 was added for 2hrs before cell collection. (e) IB analysis of WCL derived from WT or MERTK-deleted RCC4 cells treated with 60 μM PV for 30 min. (f) IB analysis of WCL derived from RCC4 cells treated with 60 μM PV for 30 min. Where indicated, BKM120 (200 nM) or IPI549 (500 nM) were added for 10 hrs before cells were stimulated with PV.

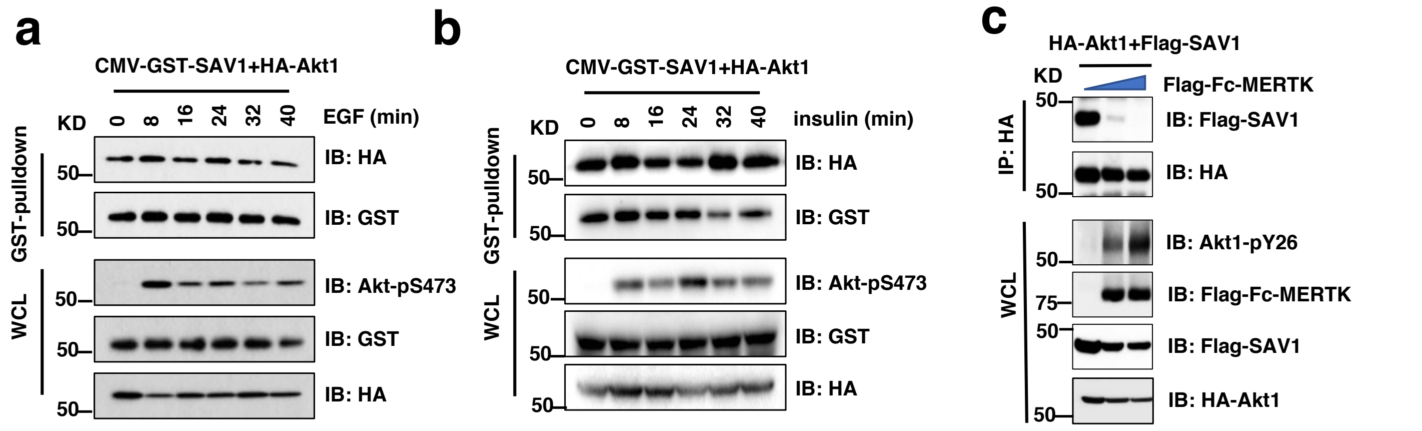

Supplementary Figure 9. Growth signaling does not regulate Akt binding with SAV1. (a-b) IB analysis of GST-pulldowns and WCL derived HeLa cells transfected with GST-SAV1 and HA-Akt1 constructs. Where indicated, cells were serum starved for 10 hrs followed by EGF (100 ng/ml) (a) or insulin (100 nM) (b) stimulation for indicated periods. (c) IB analysis of HA-IPs and WCL derived from HEK293 cells transfected with indicated constructs.

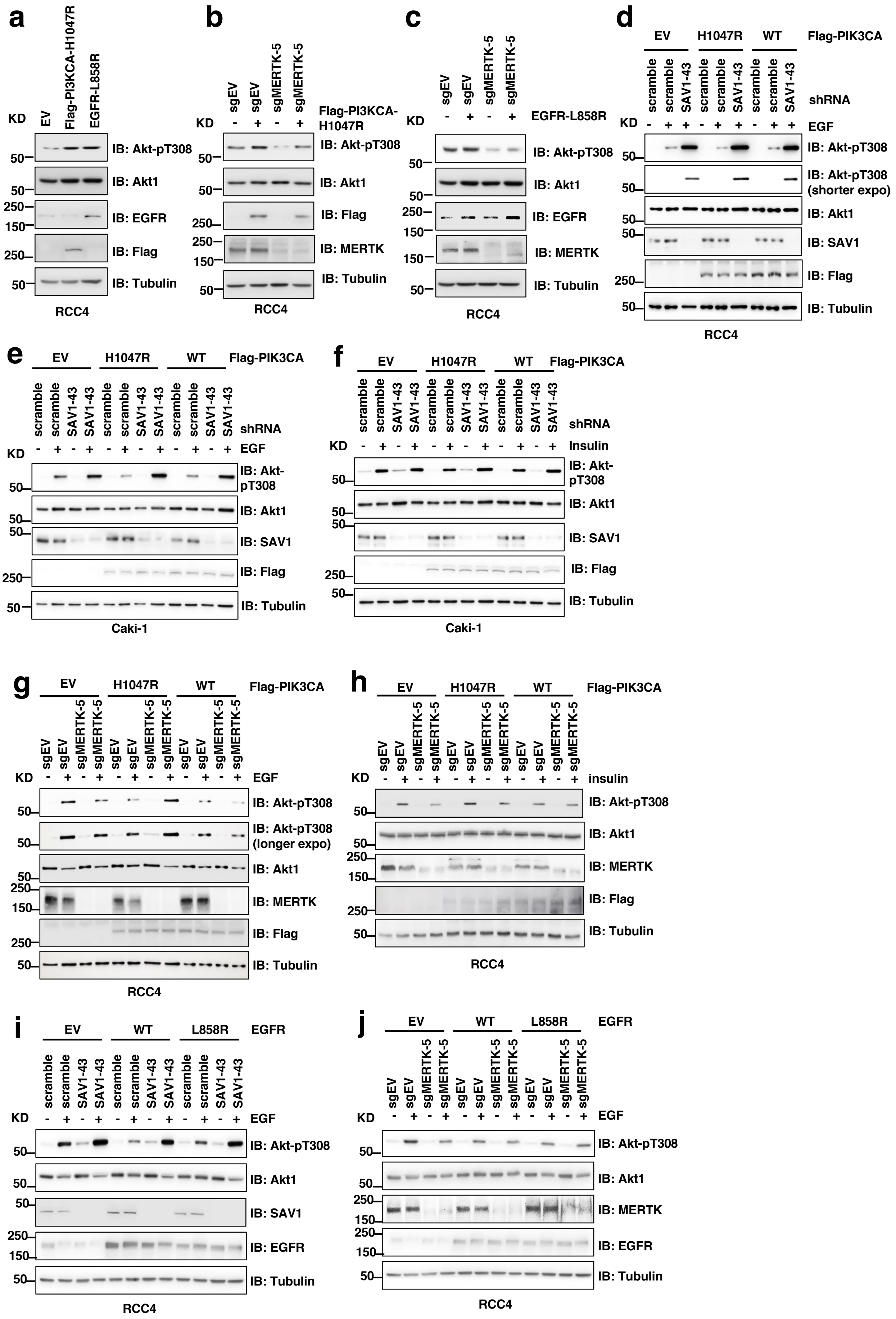
a
KD
EV
Flag-PI3KCA-H1047R
EGFR-L858R
IB: Akt-pT308
IB: Akt1
IB: EGFR
IB: Flag
IB: Tubulin
RCC4
b
sgEV
sgEV
sgMERTK-5
sgMERTK-5
Flag-PI3KCA-H1047R
IB: Akt-pT308
IB: Akt1
IB: Flag
IB: MERTK
IB: Tubulin
RCC4
c
EGFR-L858R
IB: Akt-pT308
IB: Akt1
IB: EGFR
IB: MERTK
IB: Tubulin
RCC4
d
EV
H1047R
WT
Flag-PIK3CA
scramble
SAV1-43
shRNA
EGF
IB: Akt-pT308
IB: Akt-pT308 (shorter expo)
IB: Akt1
IB: SAV1
IB: Flag
IB: Tubulin
RCC4
e
IB: Akt-pT308
IB: Akt1
IB: SAV1
IB: Flag
IB: Tubulin
Caki-1
f
Insulin
IB: Akt-pT308
IB: Akt1
IB: SAV1
IB: Flag
IB: Tubulin
Caki-1
g
IB: Akt-pT308
IB: Akt-pT308 (longer expo)
IB: Akt1
IB: MERTK
IB: Flag
IB: Tubulin
RCC4
h
insulin
IB: Akt-pT308
IB: Akt1
IB: MERTK
IB: Flag
IB: Tubulin
RCC4
i
EV
WT
L858R
EGFR
IB: Akt-pT308
IB: Akt1
IB: SAV1
IB: EGFR
IB: Tubulin
RCC4
j
IB: Akt-pT308
IB: Akt1
IB: MERTK
IB: EGFR
IB: Tubulin
RCC4

Supplementary Figure 10. Expression of constitutively active PI3K or EGFR mutants cannot compensate for MERTK loss in activating Akt. (a-c) IB analysis of WCL derived from indicated RCC4 cells transfected with indicated DNA constructs to demonstrate that PIK3CA and EGFR oncogenic mutations can activate Akt at basal levels. (d-e) IB analysis of WCL derived from indicated RCC4 (d) or Caki-1 (e) cells transfected with indicated DNA constructs. Where indicated, cells were serum starved overnight and stimulated with 100 nM EGF for 10 min before cell collection. (f) IB analysis of WCL derived from indicated Caki-1 cells transfected with indicated DNA constructs. Where indicated, cells were serum starved overnight and stimulated with 100 ng/ml insulin for 30 min before cell collection. (g-h) IB analysis of WCL derived from indicated RCC4 cells transfected with indicated DNA constructs. Where indicated, cells were serum starved overnight and stimulated with either 100 nM EGF for 10 min (g) or 100 ng/ml insulin for 30 min (h) before cell collection. (i-j) IB analysis of WCL derived from indicated RCC4 cells transfected with indicated DNA constructs. Where indicated, cells were serum starved overnight and stimulated with 100 nM EGF for 10 min before cell collection.

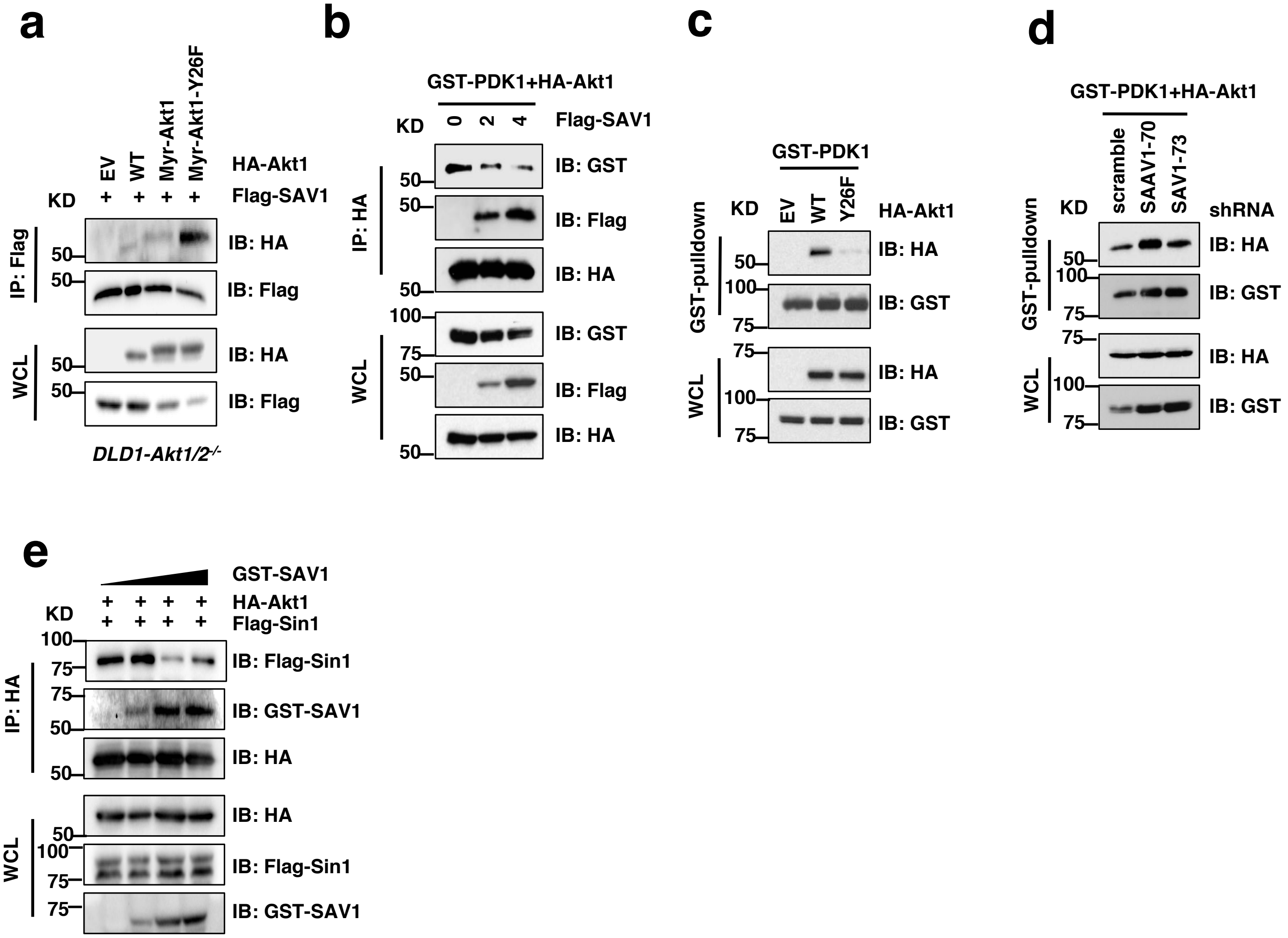

Supplementary Figure 11. SAV1 binding attenuates Akt1 interaction with PDK1 and mTORC2. (a) IB analysis of Flag-IP and WCL derived from *DLD1-Akt1/2*$^{-/-}$ cells transfected with indicated DNA constructs. (b) IB analysis of HA-IP and WCL derived from HEK293 cells transfected with indicated DNA constructs. (c) IB analyses of GST-pulldowns and WCL derived from HEK293 cells transfected with indicated DNA constructs. (d) IB analyses of GST-pulldowns and WCL derived from WT or SAV1-depleted RCC4 cells transfected with indicated DNA constructs. (e) IB analysis of HA-IP and WCL derived from HEK293 cells transfected with indicated DNA constructs.

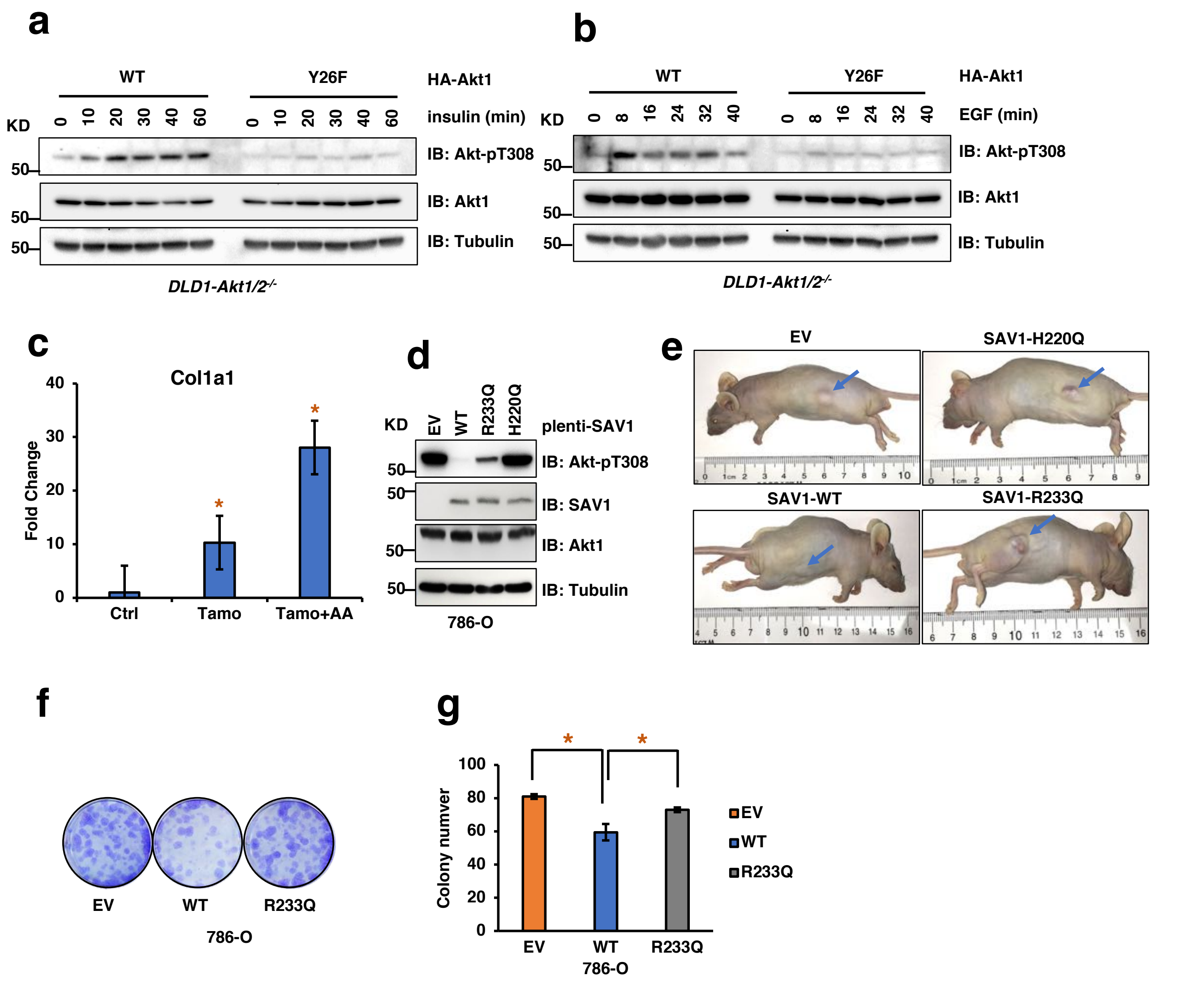

Supplementary Figure 12. SAV1 binding to Akt attenuates growth factor triggered Akt activation. (a-b) IB analyses of WCL derived from *DLD1-Akt1/2*$^{-/-}$ cells stably expressing either WT-Akt1 or Y26F-Akt1 by lenti-viral infection. Where indicated, cells were serum starved for 10 hrs and stimulated by either 100 nM insulin (a) or 100 ng/ml EGF (b) for indicated time periods. (c) Analyses of mRNA changes in Col1a1 fibrosis genes in *SAV1*^{KS} mouse kidneys receiving indicated treatments. (d) IB analysis of WCL derived from 786-O cells stably expressing indicated SAV1 constructs by lenti-viral infection and selected in 10 µg/ml blasticidin for 72 hours to eliminate non-infected cells before cell collection. (e) Representation tumor images for mouse xenograft experiments using cells generated in (d). (f-g) 600 cells from (d) were plated into 6-well plates and 21 days later cells were fixed, stained with crystal violet (f) and quantified (g).

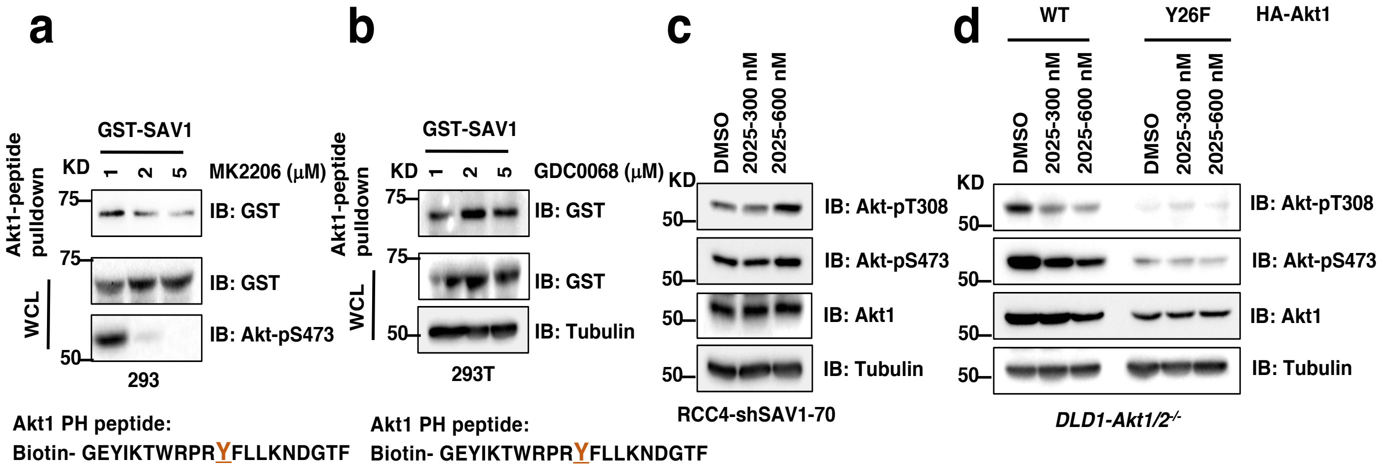

Supplementary Figure 13. MERTK inhibition governed Akt inactivation requires SAV1. (a-b) Akt1 peptide pull-down assays to indicate that an Akt allosteric inhibitor, MK2206 (a) but not an Akt-ATP analog inhibitor, GDC0068 (b) competes with SAV1 to bind the Akt PH domain. (c) IB analysis of WCL derived from RCC4 cells depleted of endogenous SAV1 and treated with indicated doses of UNC2025 for 2 hrs. (d) IB analyses of WCL derived from *DLD1-Akt1/2*$^{-/-}$ cells stably expressing either WT-Akt1 or Y26F-Akt1 by lenti-viral infection that were treated with indicated doses of UNC2025 for 2 hrs before cell collection.

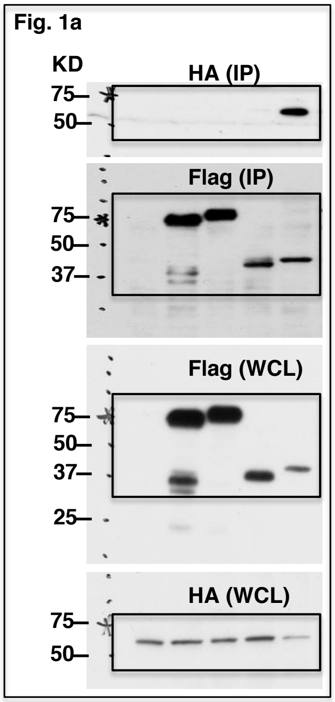
Fig. 1a
KD
HA (IP)
75
50
Flag (IP)
75
50
37
Flag (WCL)
75
50
37
25
HA (WCL)
75
50

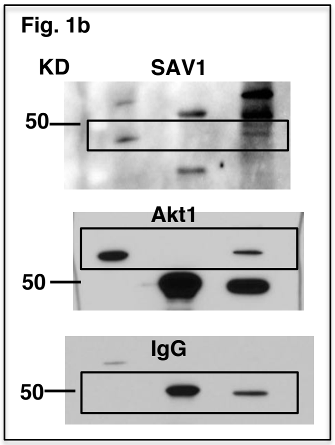
Fig. 1b
KD
SAV1
50
Akt1
50
IgG
50

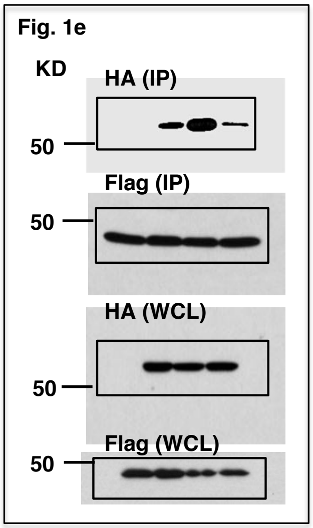
Fig. 1e
KD
HA (IP)
50
Flag (IP)
50
HA (WCL)
50
Flag (WCL)
50

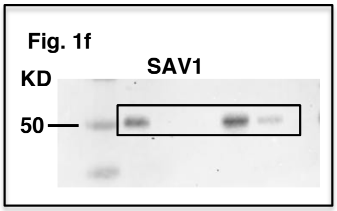
Fig. 1f
KD
SAV1
50

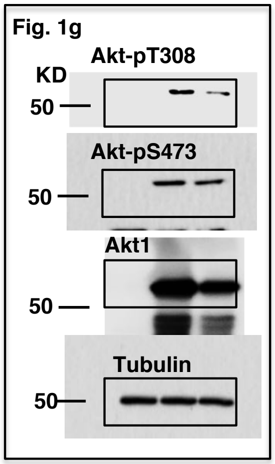
Fig. 1g
Akt-pT308
KD
50
Akt-pS473
50
Akt1
50
Tubulin
50

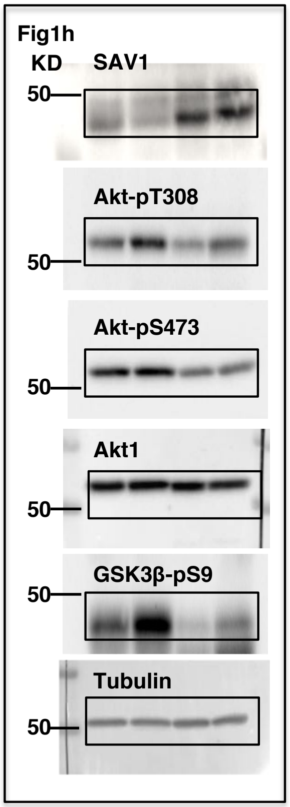
Fig1h
KD
SAV1
50
Akt-pT308
50
Akt-pS473
50
Akt1
50
GSK3β-pS9
50
Tubulin
50

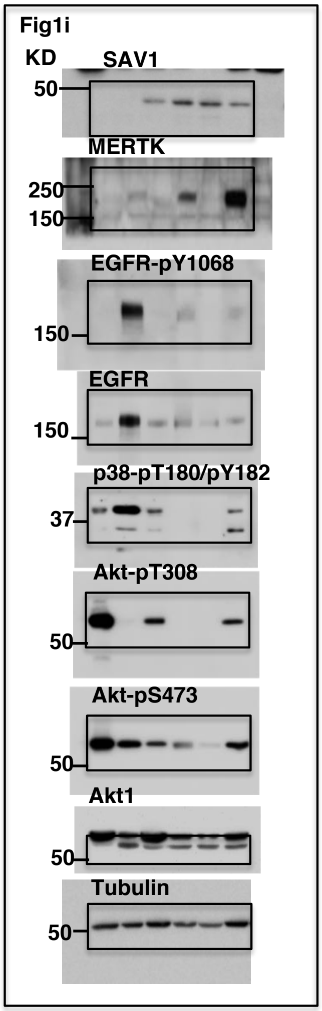
Fig1i
KD
SAV1
50
MERTK
250
150
EGFR-pY1068
150
EGFR
150
p38-pT180/pY182
37
Akt-pT308
50
Akt-pS473
50
Akt1
50
Tubulin
50

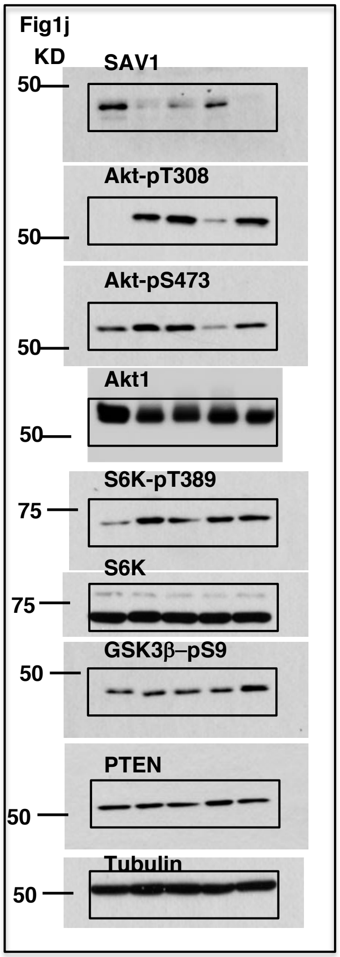
Fig1j
KD
SAV1
50
Akt-pT308
50
Akt-pS473
50
Akt1
50
S6K-pT389
75
S6K
75
GSK3β–pS9
50
PTEN
50
Tubulin
50

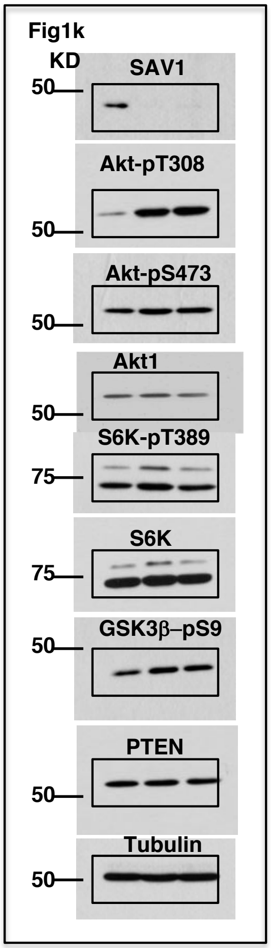
Fig1k
KD
SAV1
50
Akt-pT308
50
Akt-pS473
50
Akt1
50
S6K-pT389
75
S6K
75
GSK3β–pS9
50
PTEN
50
Tubulin
50

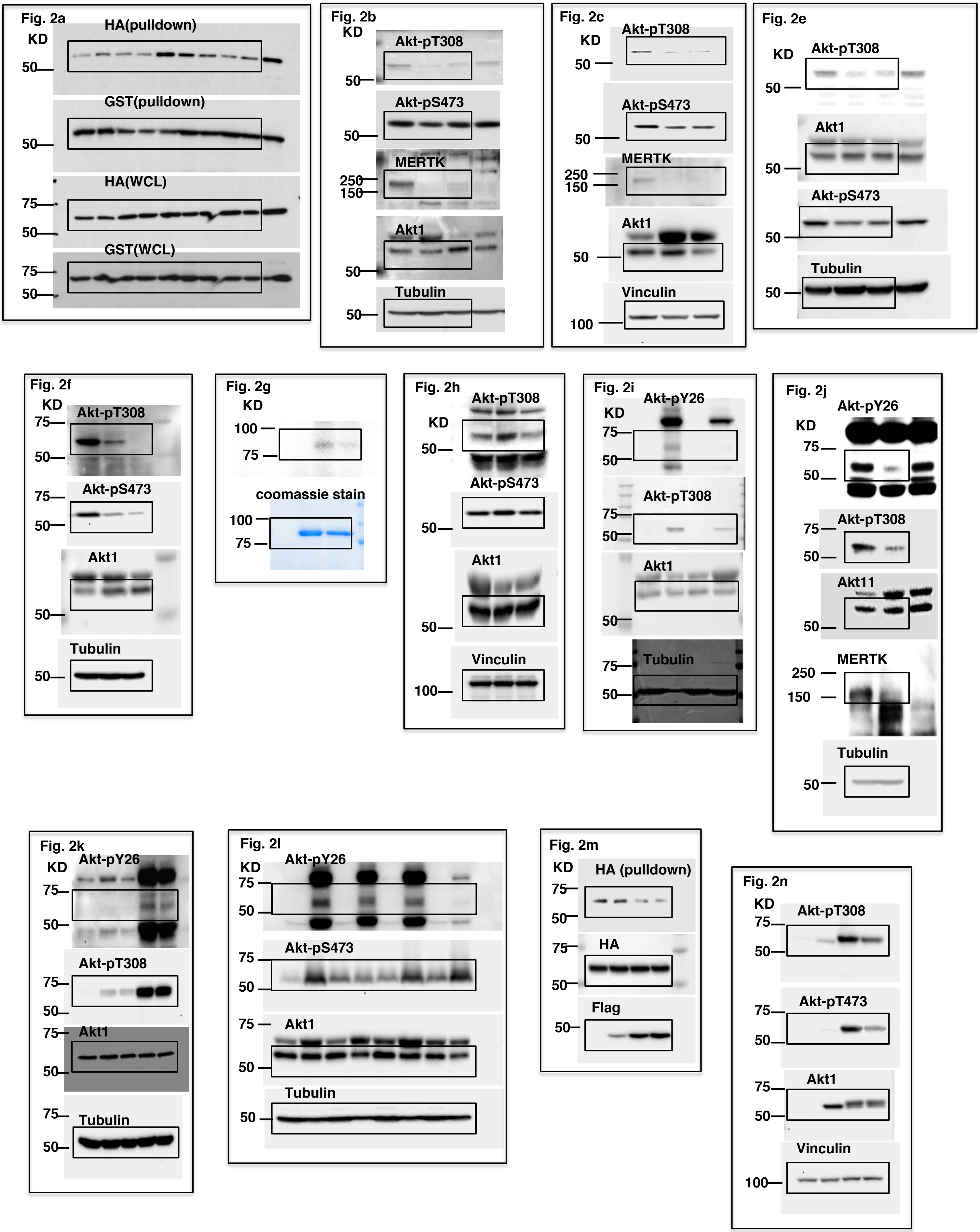
Fig. 2a
KD
HA(pulldown)
GST(pulldown)
HA(WCL)
GST(WCL)
50
75
Fig. 2b
KD
Akt-pT308
Akt-pS473
MERTK
Akt1
Tubulin
250
150
50
Fig. 2c
KD
Akt-pT308
Akt-pS473
MERTK
Akt1
Vinculin
100
Fig. 2e
KD
Akt-pT308
Akt1
Akt-pS473
Tubulin
Fig. 2f
KD
Akt-pT308
Akt-pS473
Akt1
Tubulin
Fig. 2g
KD
coomassie stain
Fig. 2h
KD
Akt-pT308
Akt-pS473
Akt1
Vinculin
Fig. 2i
KD
Akt-pY26
Akt-pT308
Akt1
Tubulin
Fig. 2j
KD
Akt-pY26
Akt-pT308
Akt11
MERTK
Tubulin
Fig. 2k
KD
Akt-pY26
Akt-pT308
Akt1
Tubulin
Fig. 2l
KD
Akt-pY26
Akt-pS473
Akt1
Tubulin
Fig. 2m
KD
HA (pulldown)
HA
Flag
Fig. 2n
KD
Akt-pT308
Akt-pT473
Akt1
Vinculin

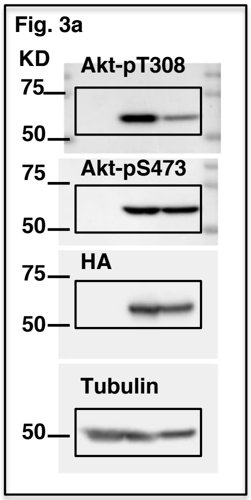
Fig. 3a
KD
Akt-pT308
75
50
Akt-pS473
75
50
HA
75
50
Tubulin
50

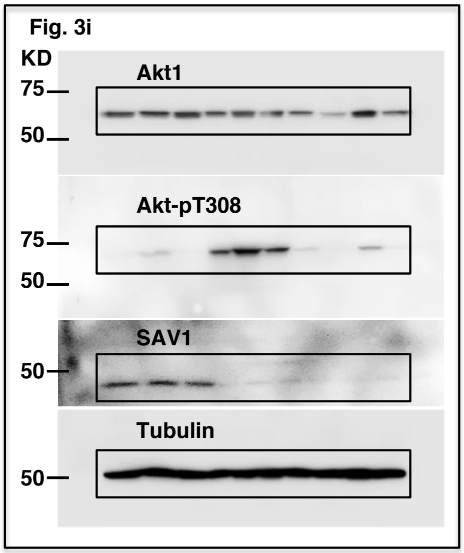
Fig. 3i
KD
Akt1
75
50
Akt-pT308
75
50
SAV1
50
Tubulin
50

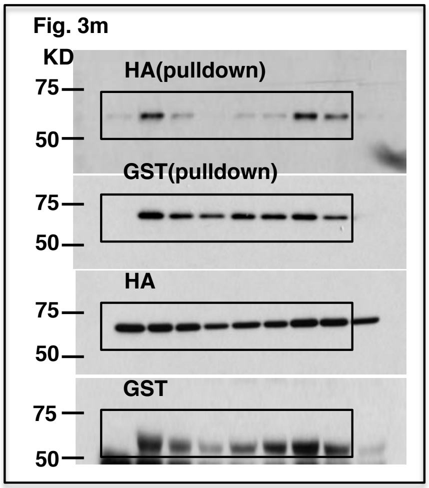
Fig. 3m
KD
HA(pulldown)
75
50
GST(pulldown)
75
50
HA
75
50
GST
75
50

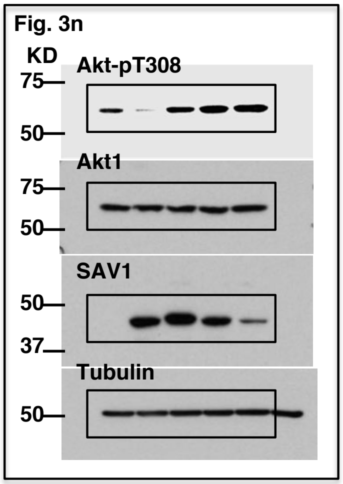
Fig. 3n
KD
Akt-pT308
75
50
Akt1
75
50
SAV1
50
37
Tubulin
50

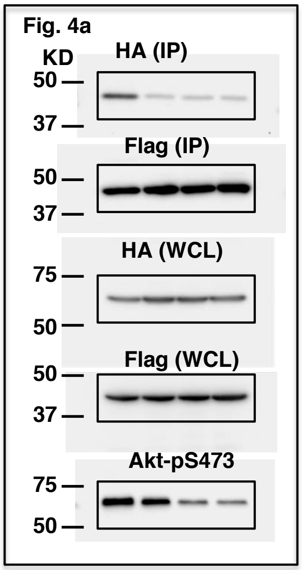
Fig. 4a
KD
HA (IP)
50
37
Flag (IP)
50
37
HA (WCL)
75
50
Flag (WCL)
50
37
Akt-pS473
75
50

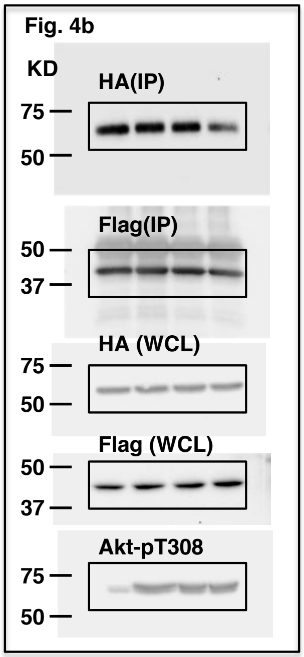
Fig. 4b
KD
HA(IP)
75
50
Flag(IP)
50
37
HA (WCL)
75
50
Flag (WCL)
50
37
Akt-pT308
75
50

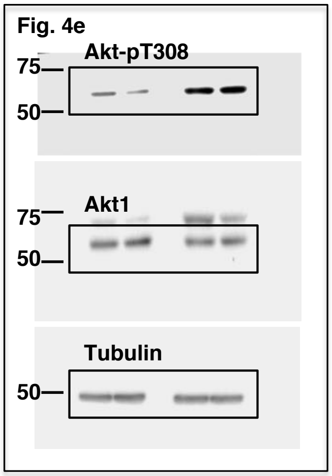
Fig. 4e
Akt-pT308
75
50
Akt1
75
50
Tubulin
50

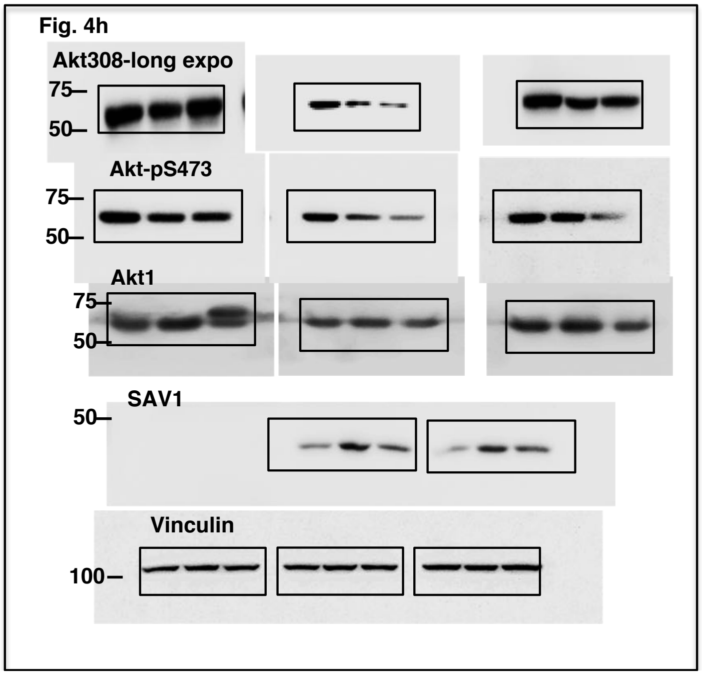
Fig. 4h
Akt308-long expo
75
50
Akt-pS473
75
50
Akt1
75
50
SAV1
50
Vinculin
100

Fig. S1b

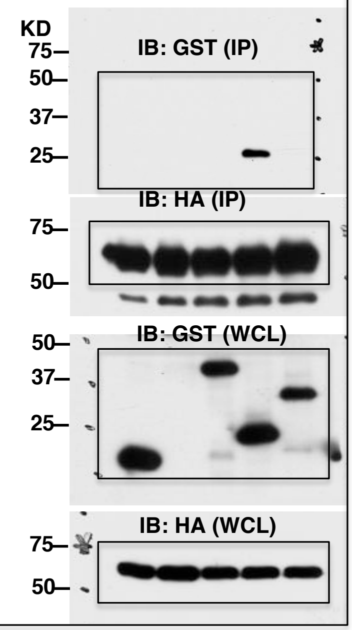

Fig. S1c

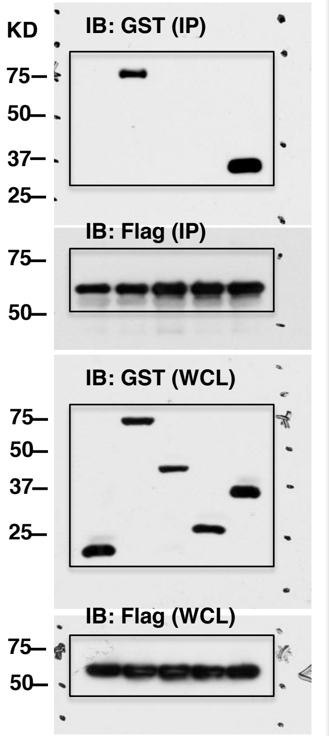

Fig. S1e

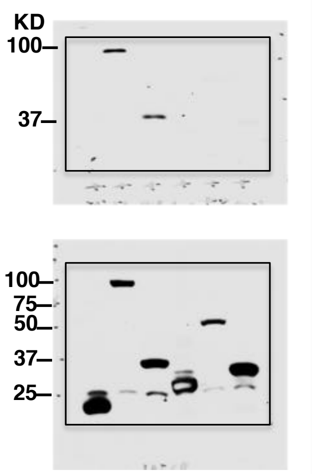

Fig. S1f

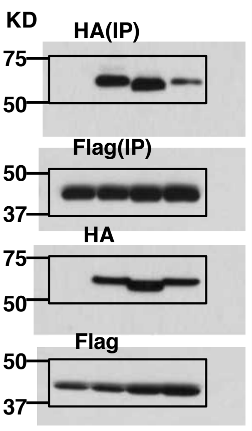

Fig. S1h

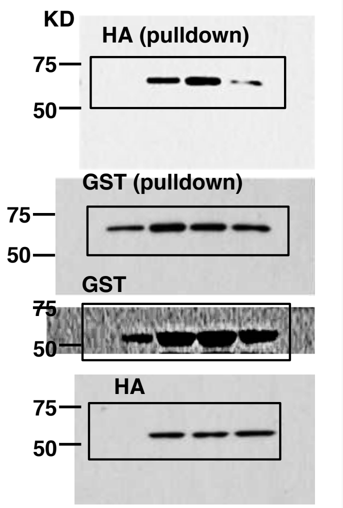

Fig. S1i

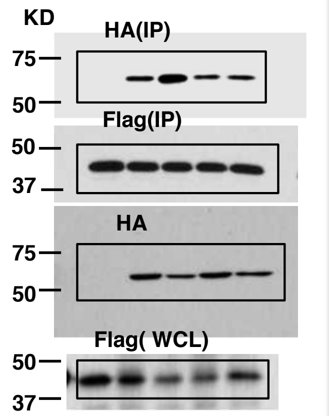

Fig. S1l

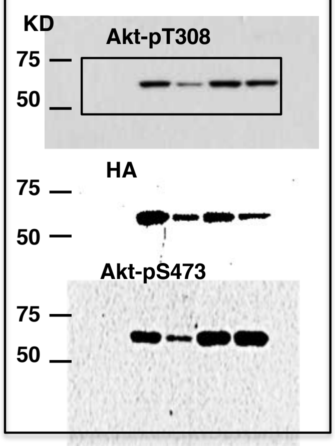

Fig. S1m

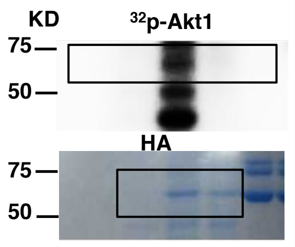

Fig. S2b

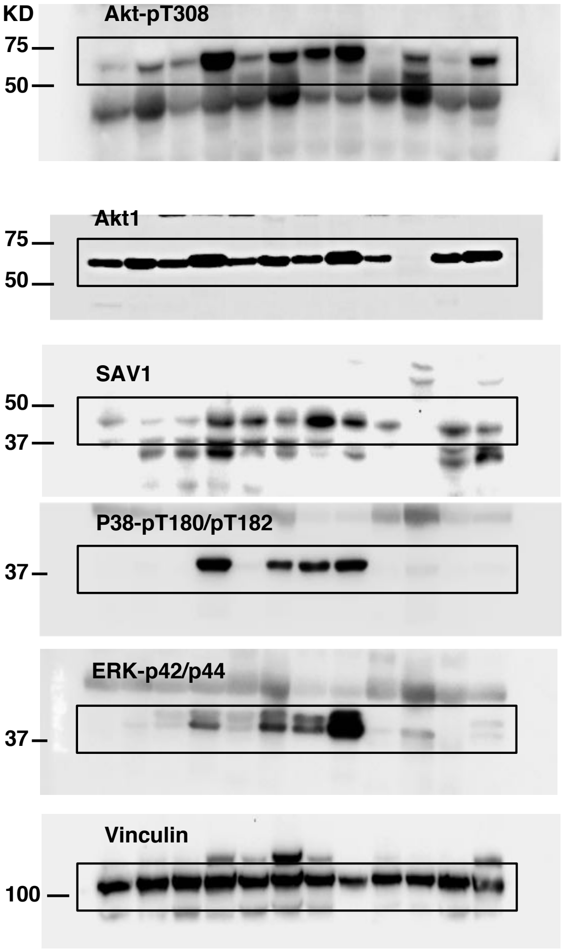

Fig. S3a

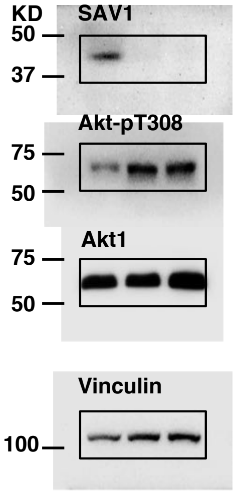

Fig. S3b

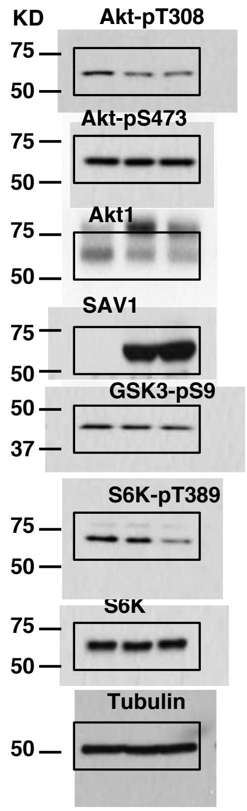

Fig. S3c

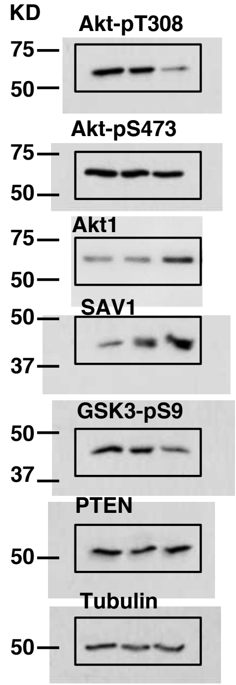

Fig. S3d

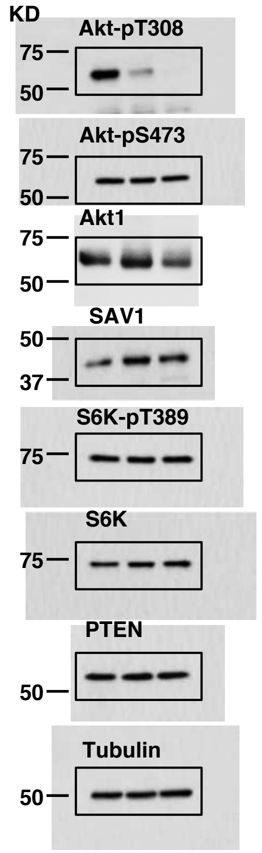

Fig. S3f

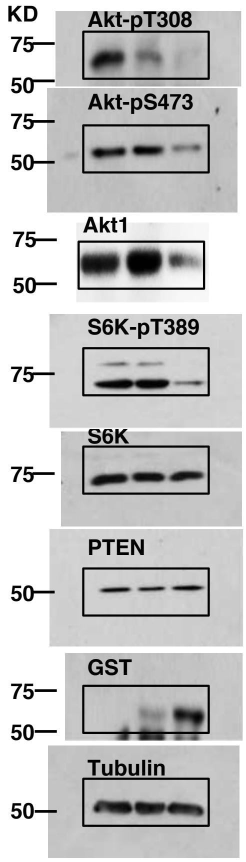

Fig. S3e

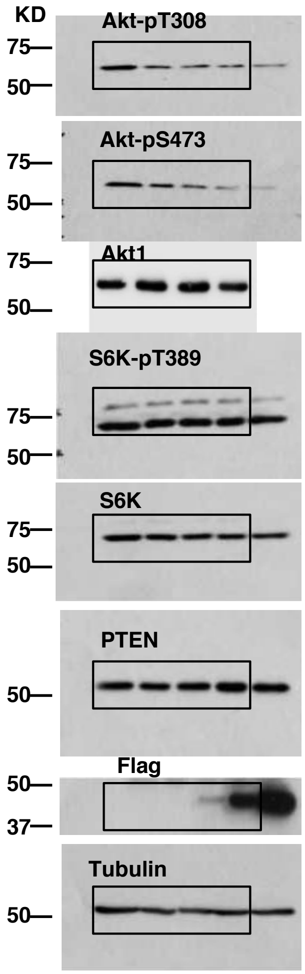

Fig. S3g

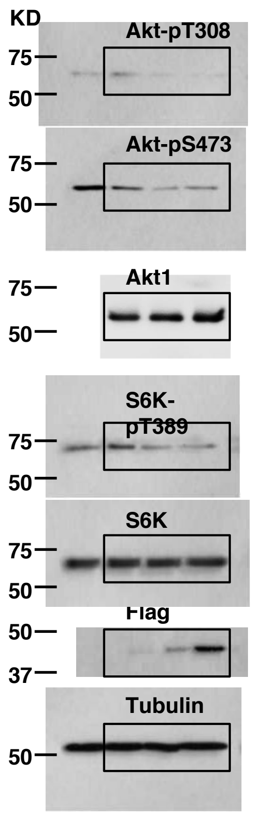

Fig. S3h

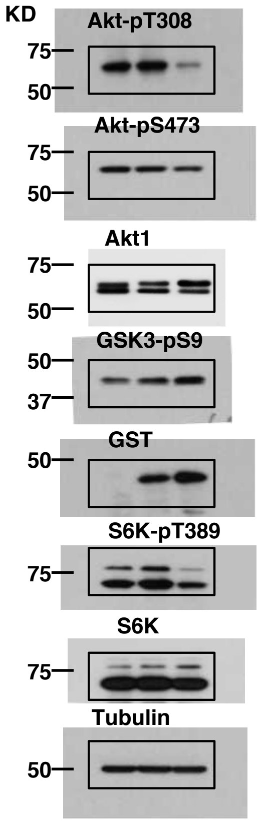

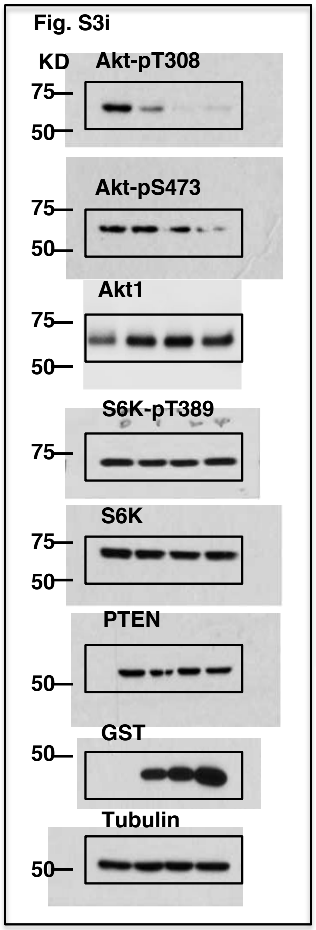
Fig. S3i
KD
Akt-pT308
75
50
Akt-pS473
75
50
Akt1
75
50
S6K-pT389
75
S6K
75
50
PTEN
50
GST
50
Tubulin
50

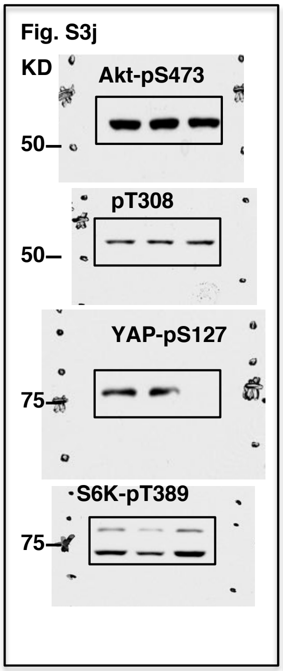
Fig. S3j
KD
Akt-pS473
50
pT308
50
YAP-pS127
75
S6K-pT389
75

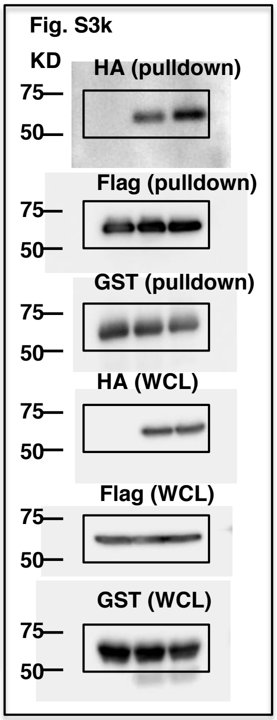
Fig. S3k
KD
HA (pulldown)
75
50
Flag (pulldown)
75
50
GST (pulldown)
75
50
HA (WCL)
75
50
Flag (WCL)
75
50
GST (WCL)
75
50

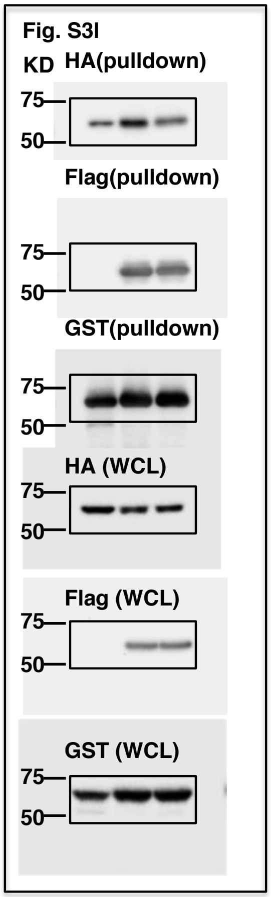
Fig. S3l
KD
HA(pulldown)
75
50
Flag(pulldown)
75
50
GST(pulldown)
75
50
HA (WCL)
75
50
Flag (WCL)
75
50
GST (WCL)
75
50

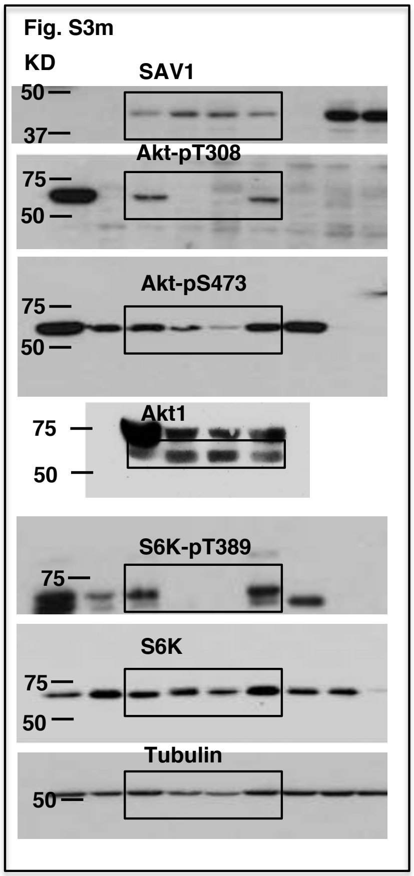
Fig. S3m
KD
SAV1
50
37
Akt-pT308
75
50
Akt-pS473
75
50
Akt1
75
50
S6K-pT389
75
S6K
75
50
Tubulin
50

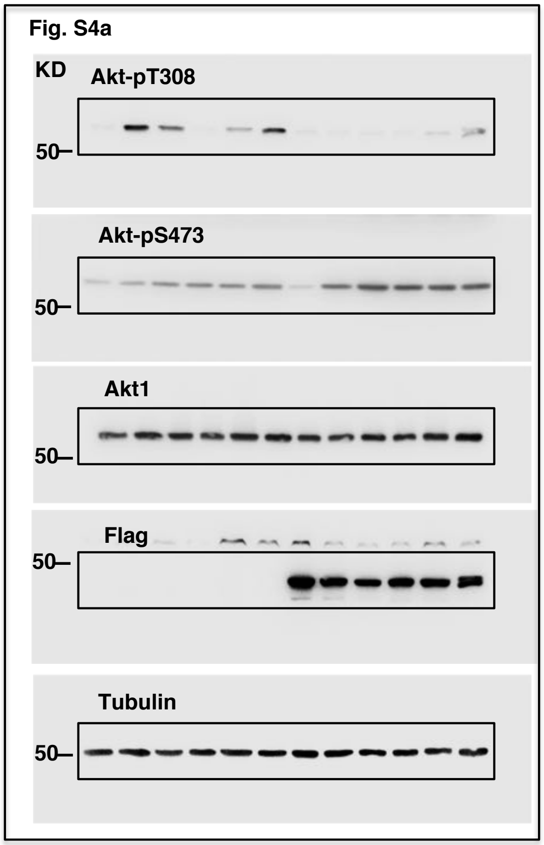
Fig. S4a
KD
Akt-pT308
50
Akt-pS473
50
Akt1
50
Flag
50
Tubulin
50

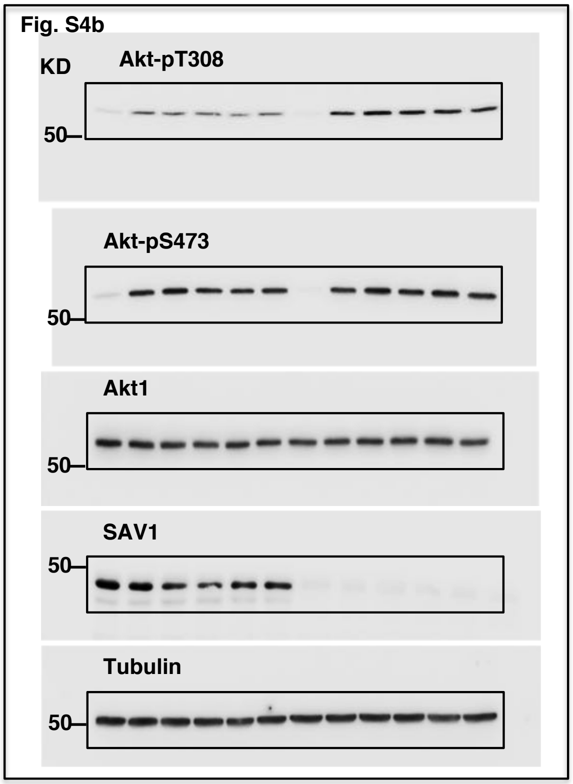
Fig. S4b
KD
Akt-pT308
50
Akt-pS473
50
Akt1
50
SAV1
50
Tubulin
50

Fig. S4c

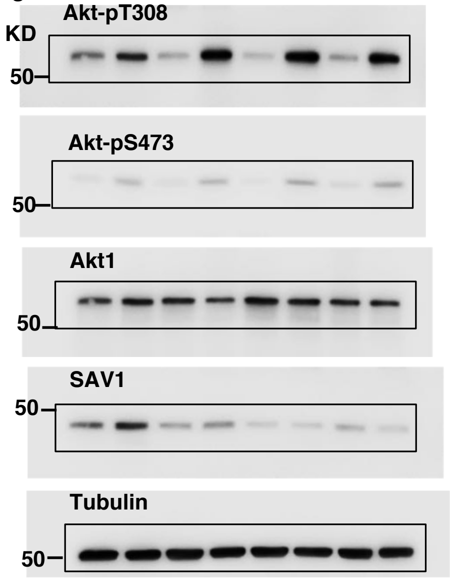

Fig. S4d

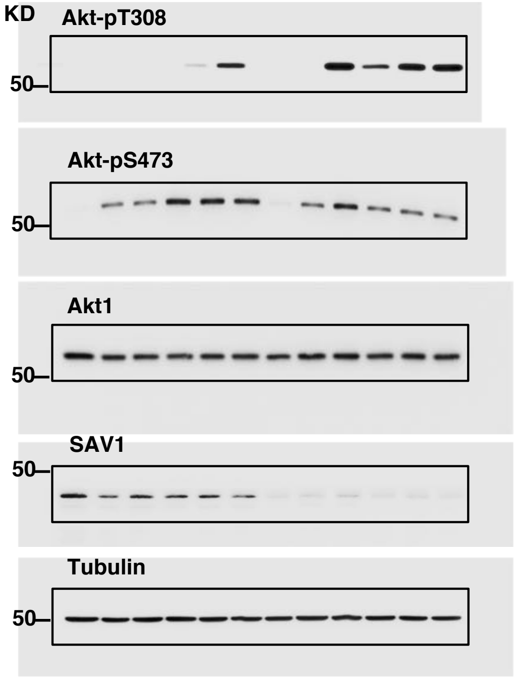

Fig. S5a

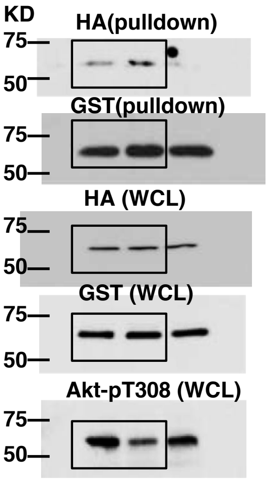

Fig. S5b

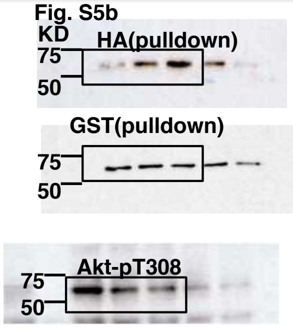

Fig. S5c

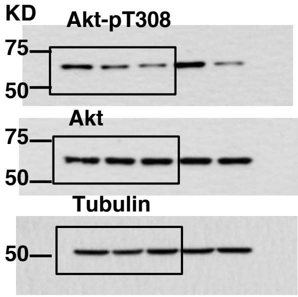

Fig. S5d

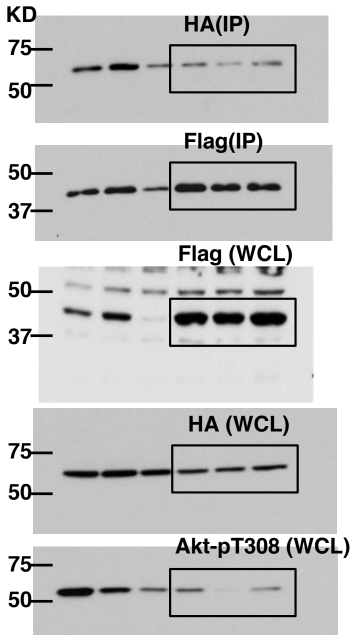

Fig. S5e

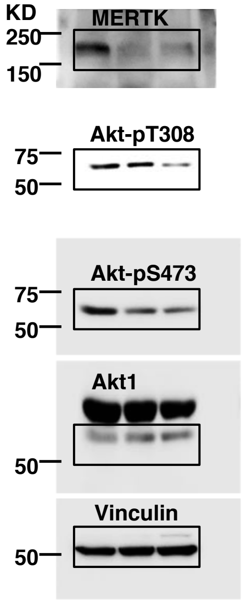

Fig. S5f

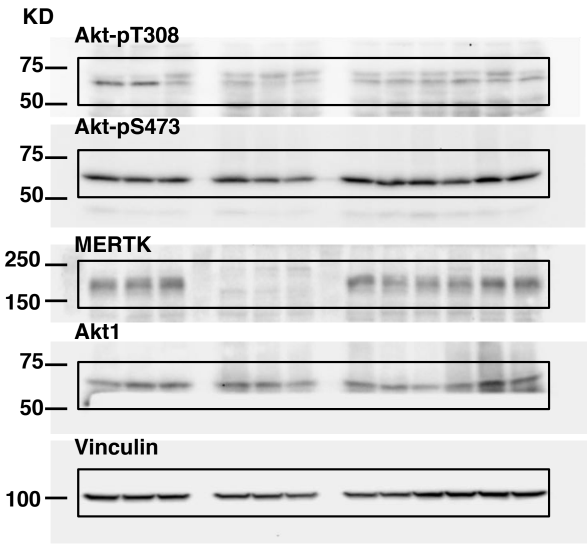

Fig. S5h

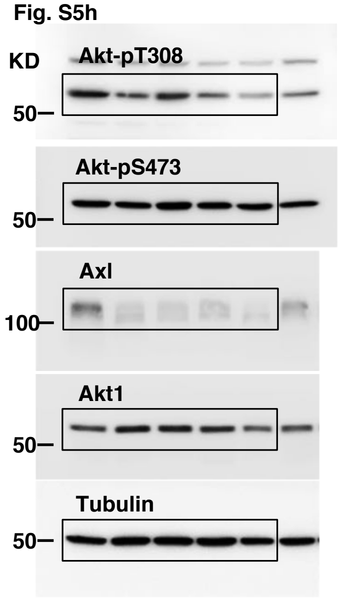

Fig. S5i

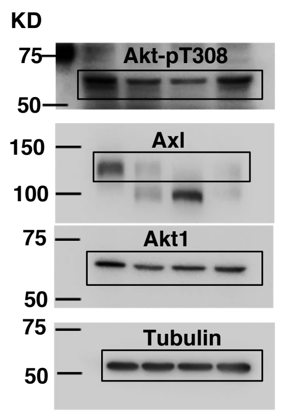

Fig. S5j

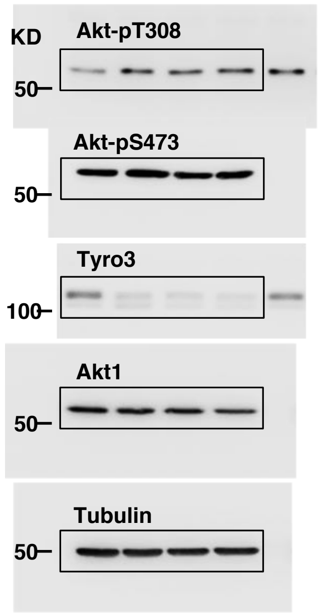

Fig. S5k

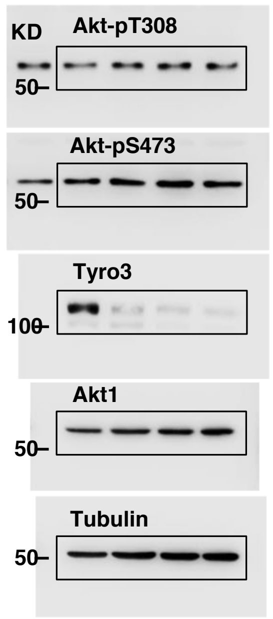

Fig. S6a

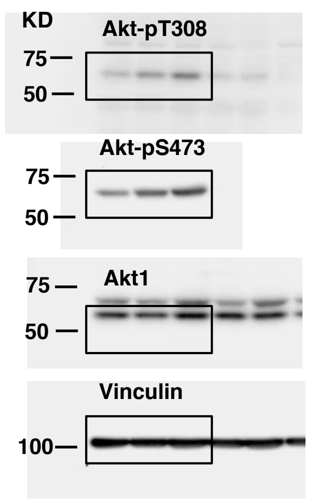

Fig. S6d

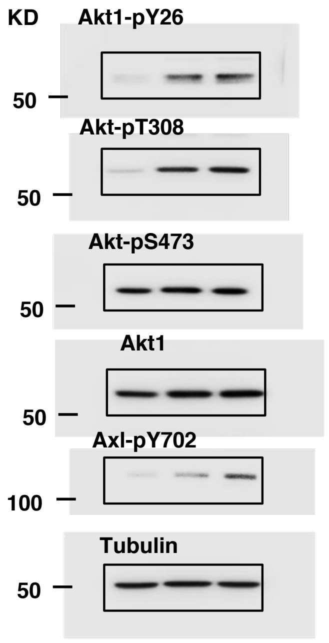

Fig. S6e

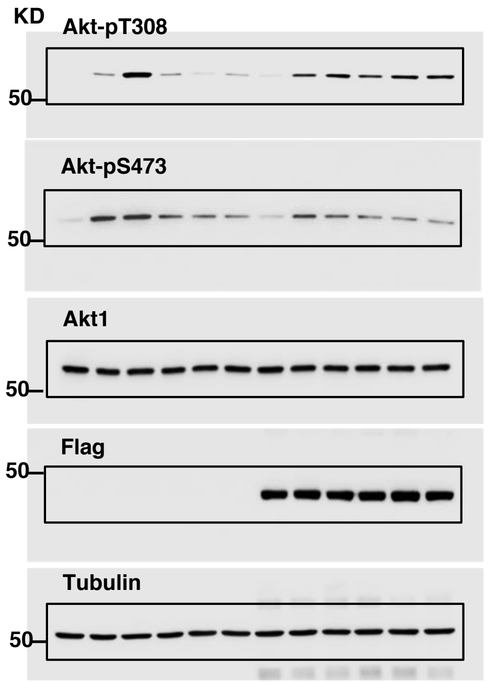

Fig. S6f

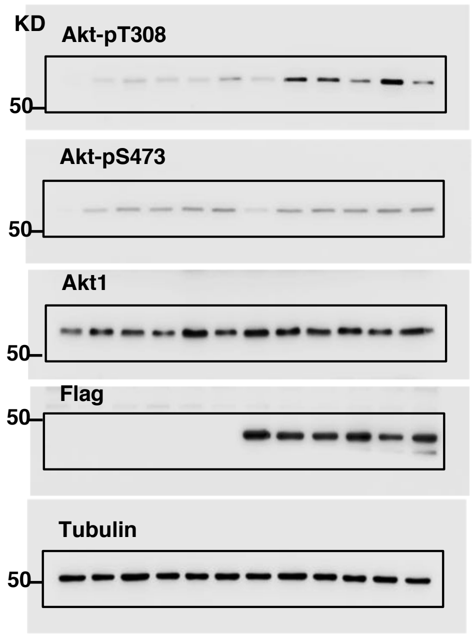

Fig. S6g

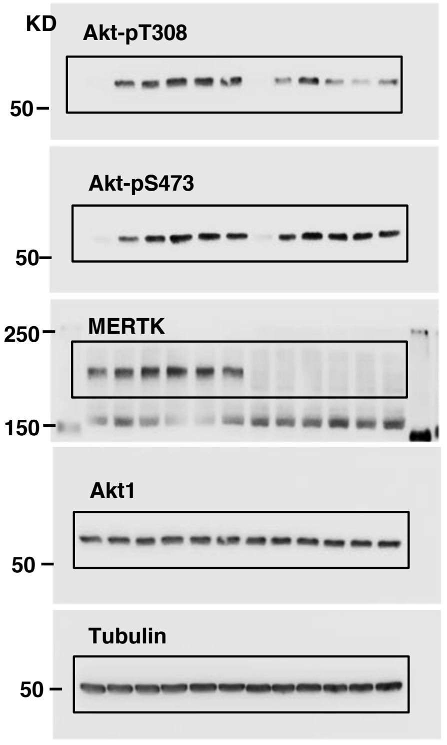

Fig. S6h

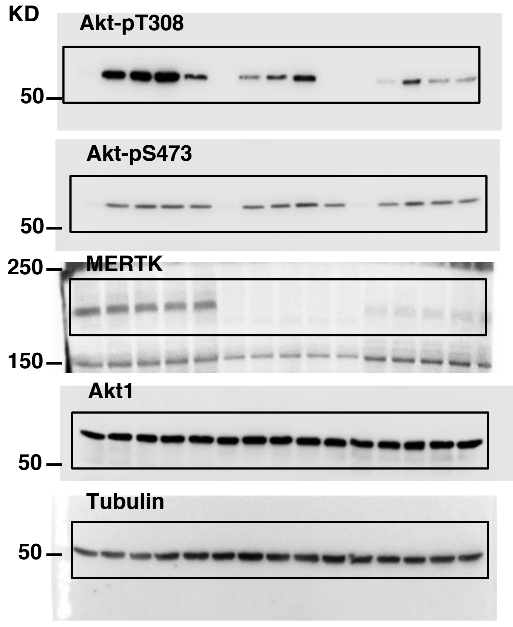

Fig. S7b

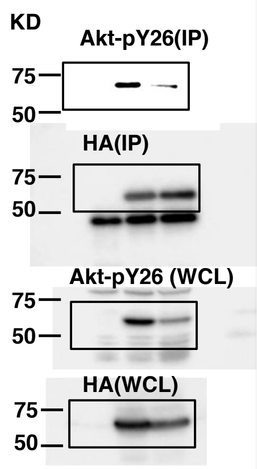

Fig. S8a

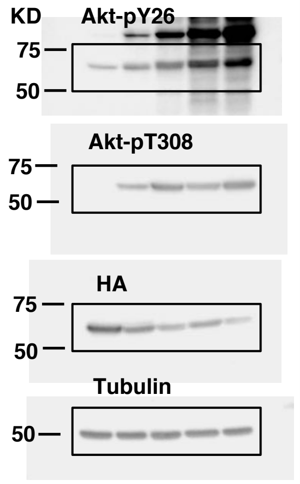

Fig. S8b

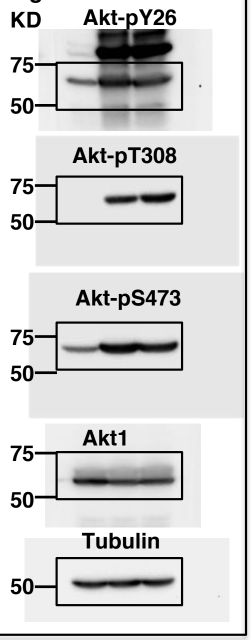

Fig. S8c

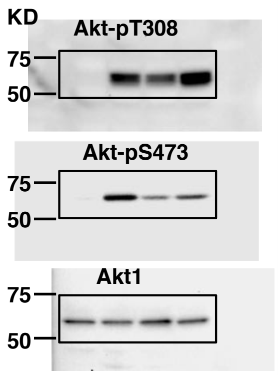

Fig. S8d

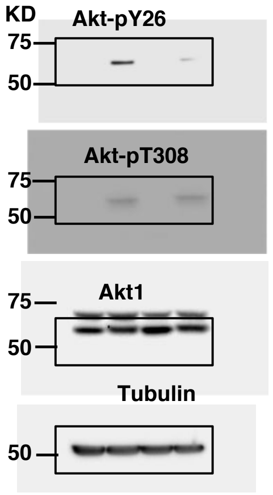

Fig. S8e
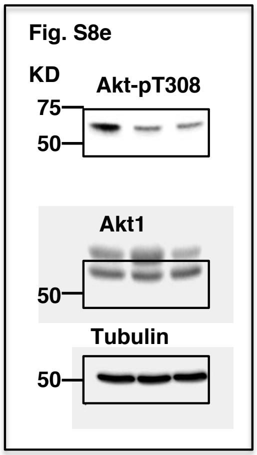

Fig. S8f
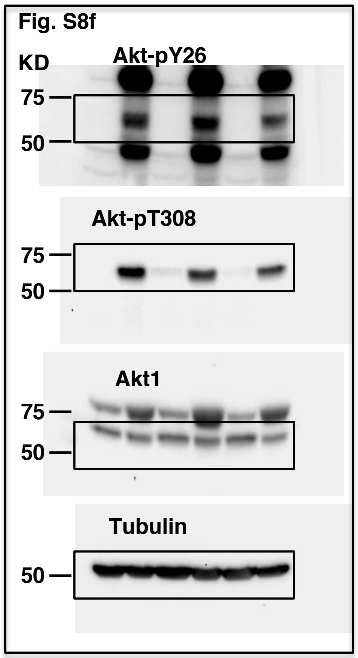

Fig. S9a
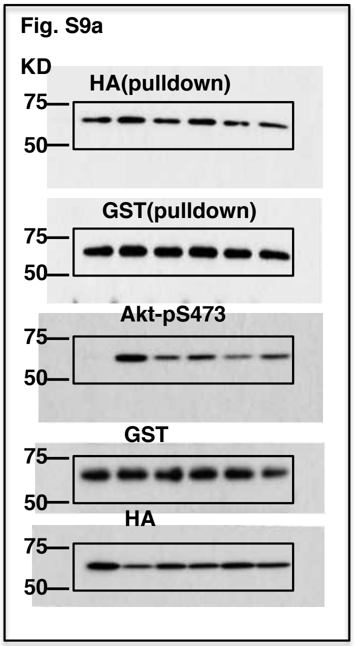

Fig. S9b
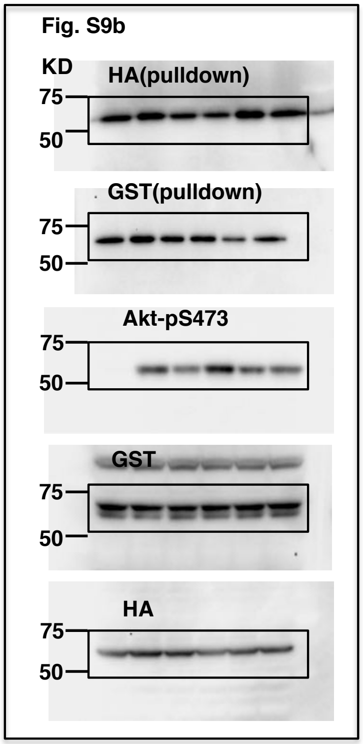

Fig. S9c
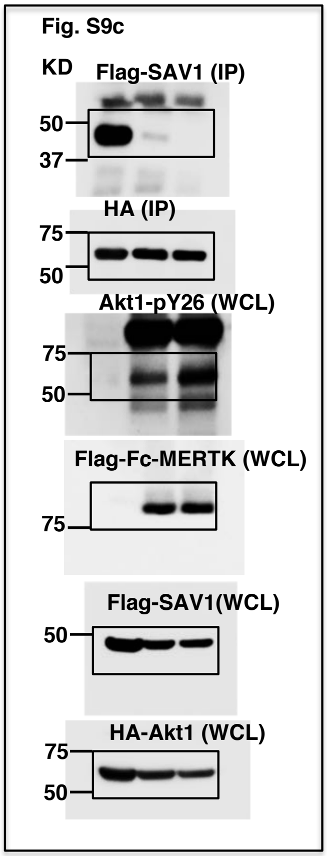

Fig. S10a
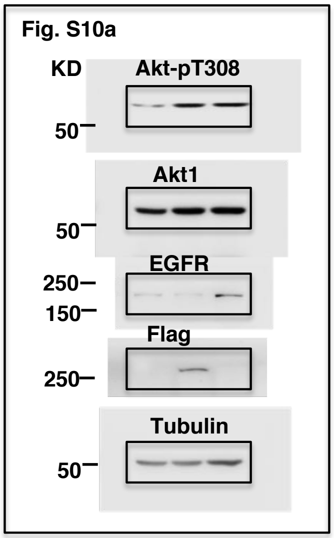

Fig. S10b
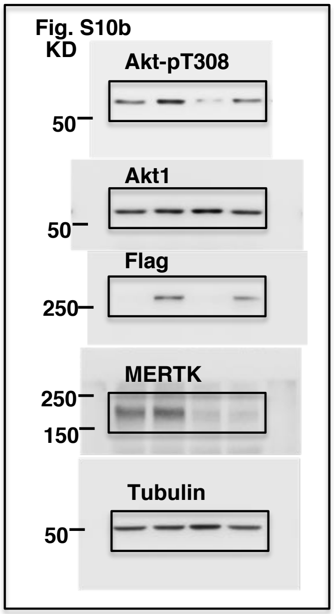

Fig. S10C
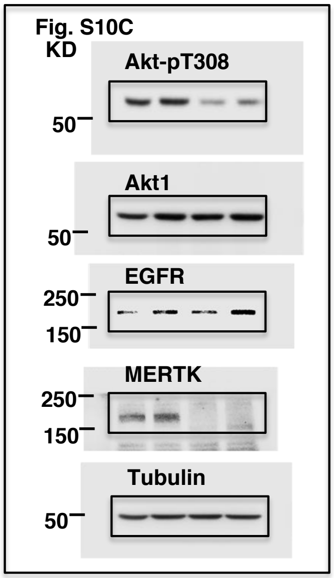

Fig. S10d

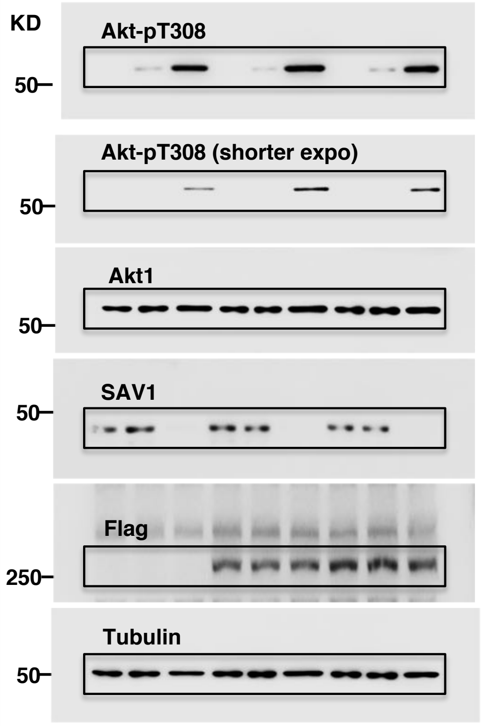

Fig. S10e

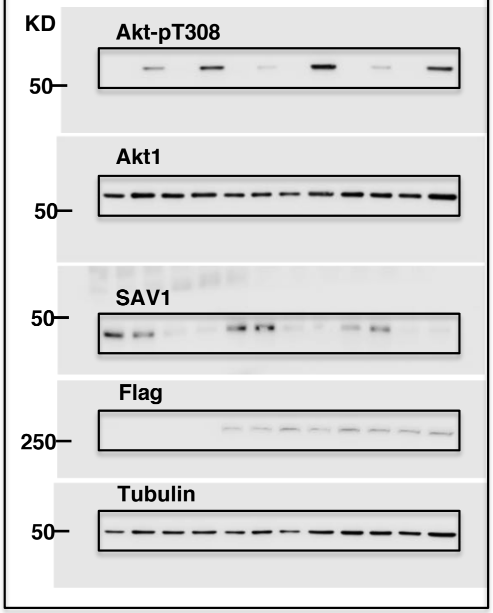

Fig. S10f

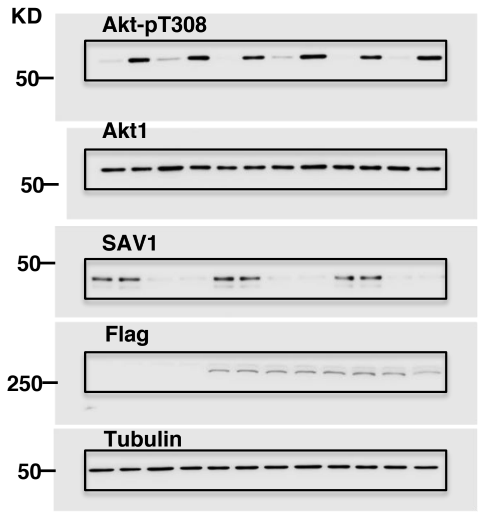

Fig. S10g

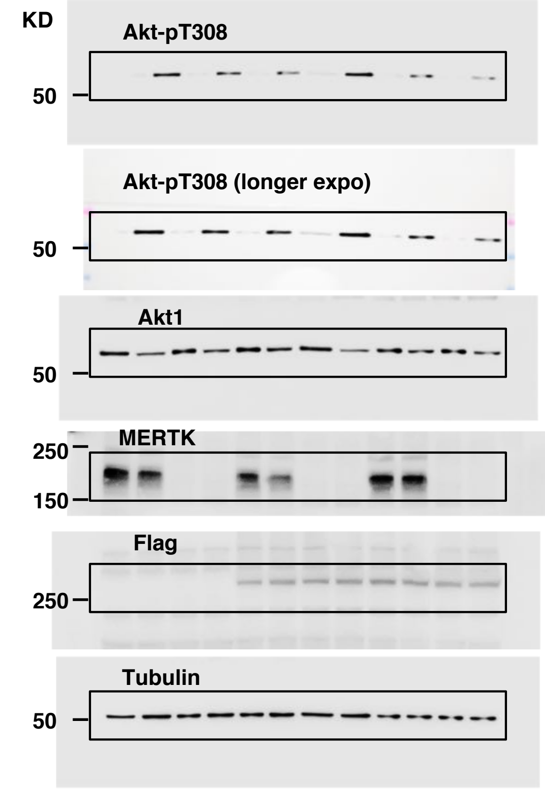

Fig. S10h

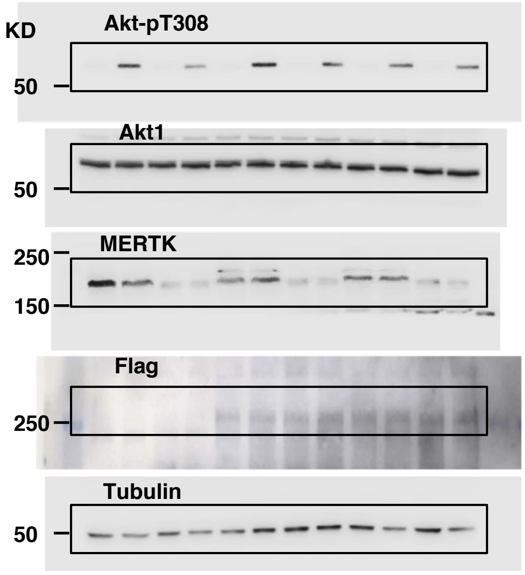

Fig. S10i

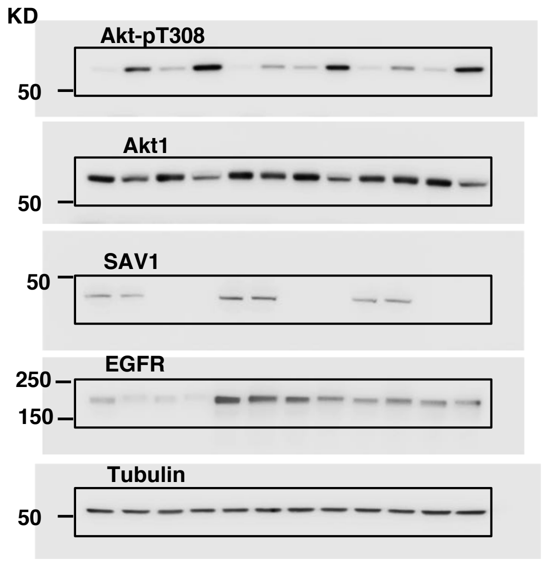

Fig. S10j

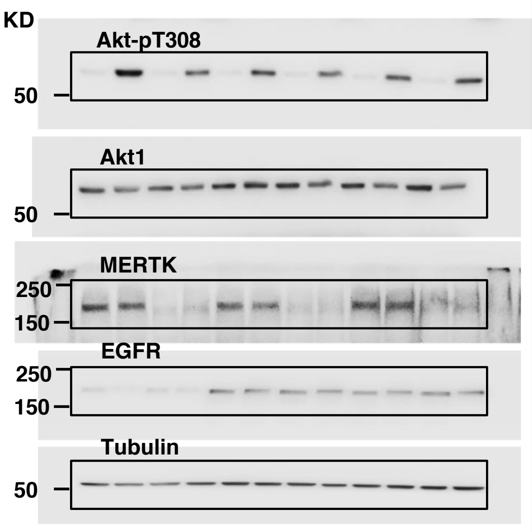

Fig. S11a

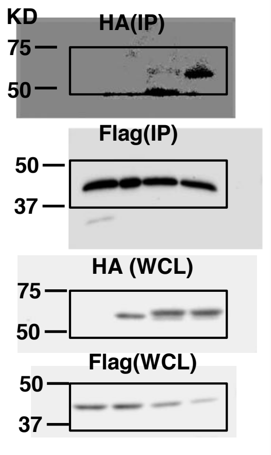

Fig. S11b

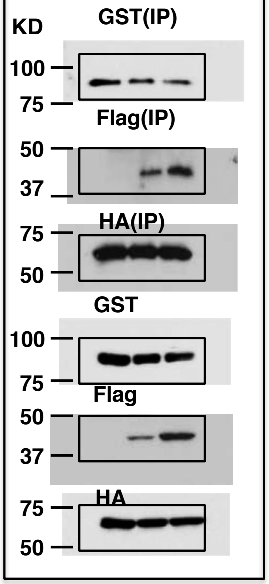

Fig. S11c

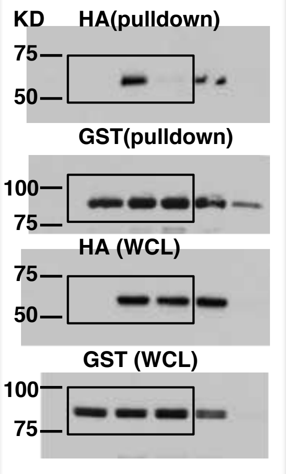

Fig. S11d

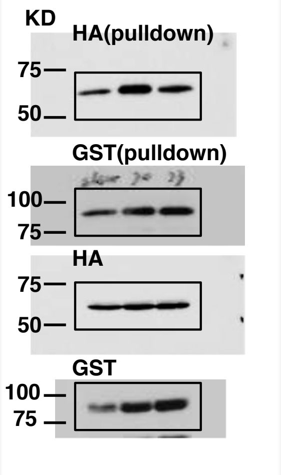

Fig. S12a

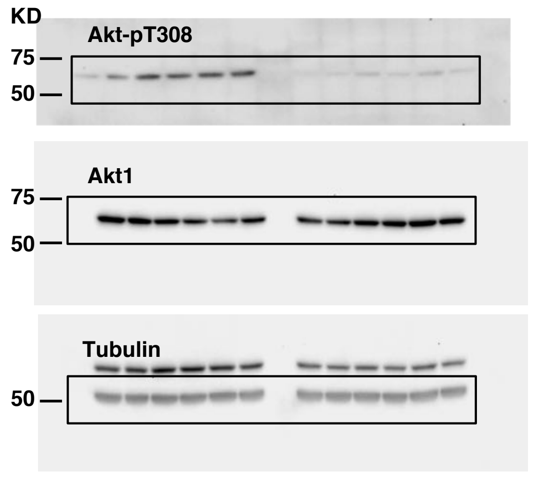

Fig. S12b

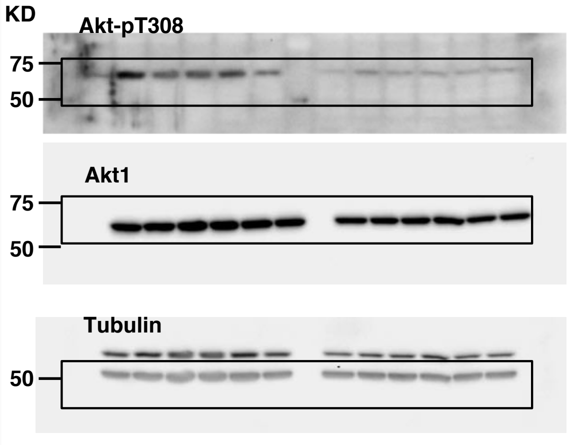

Fig. S11e

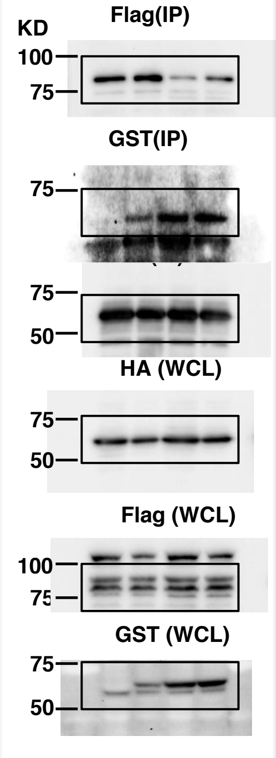

Fig. S12d

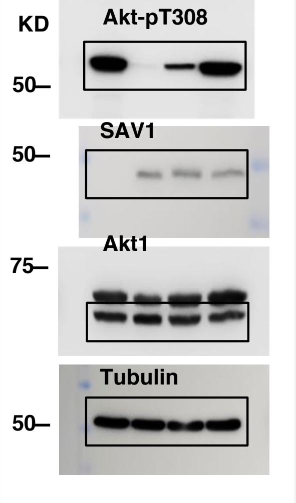

Fig. S13a

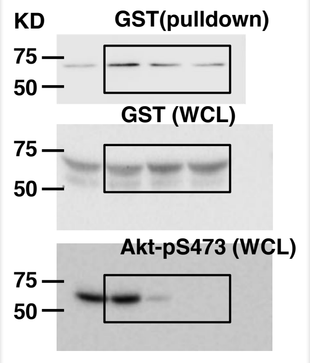

Fig. S13b

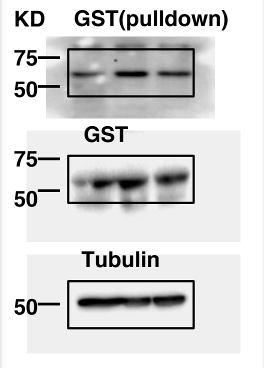

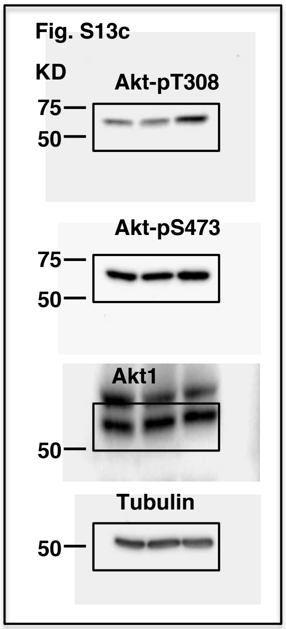
Fig. S13c
KD
Akt-pT308
75
50
Akt-pS473
75
50
Akt1
50
Tubulin
50

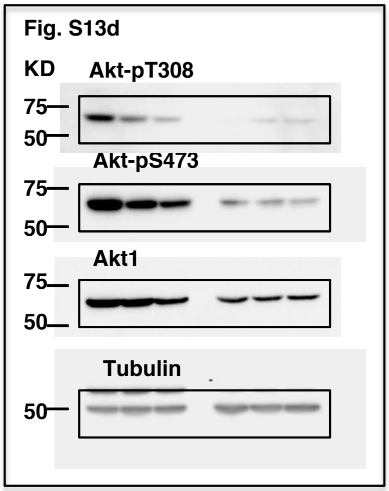
Fig. S13d
KD
Akt-pT308
75
50
Akt-pS473
75
50
Akt1
75
50
Tubulin
50

Supplementary Figure 14. Original uncropped scans for western blots.
