# Supplementary material for: MERTK mediated novel site Akt phosphorylation alleviates SAV1 suppression
Source: Nat Commun. 2019 Apr 3;10:1515. doi: 10.1038/s41467-019-09233-7 (PMC6447540; doi:10.1038/s41467-019-09233-7)
Supplement: Supplementary file 3 — Reporting Summary [file 41467_2019_9233_MOESM3_ESM.pdf]

natureresearch

Corresponding author(s): Pengda Liu

Last updated by author(s): Feb 14, 2019

Reporting Summary

Nature Research wishes to improve the reproducibility of the work that we publish. This form provides structure for consistency and transparency in reporting. For further information on Nature Research policies, see Authors & Referees and the Editorial Policy Checklist.

Statistics

For all statistical analyses, confirm that the following items are present in the figure legend, table legend, main text, or Methods section.

n/a	Confirmed	
☐	☒	The exact sample size (*n*) for each experimental group/condition, given as a discrete number and unit of measurement
☐	☒	A statement on whether measurements were taken from distinct samples or whether the same sample was measured repeatedly
☐	☒	The statistical test(s) used AND whether they are one- or two-sided<br>*Only common tests should be described solely by name; describe more complex techniques in the Methods section.*
☒	☐	A description of all covariates tested
☒	☐	A description of any assumptions or corrections, such as tests of normality and adjustment for multiple comparisons
☐	☒	A full description of the statistical parameters including central tendency (e.g. means) or other basic estimates (e.g. regression coefficient) AND variation (e.g. standard deviation) or associated estimates of uncertainty (e.g. confidence intervals)
☐	☒	For null hypothesis testing, the test statistic (e.g. *F*, *t*, *r*) with confidence intervals, effect sizes, degrees of freedom and *P* value noted<br>*Give P values as exact values whenever suitable.*
☒	☐	For Bayesian analysis, information on the choice of priors and Markov chain Monte Carlo settings
☒	☐	For hierarchical and complex designs, identification of the appropriate level for tests and full reporting of outcomes
☐	☒	Estimates of effect sizes (e.g. Cohen's *d*, Pearson's *r*), indicating how they were calculated

Our web collection on statistics for biologists contains articles on many of the points above.

Software and code

Policy information about availability of computer code

Data collection	Gen 5.1.10 software (Biotek), ClusPro server, Eris, MacPymol
Data analysis	GraphPad Prism 5 (GraphPad Software, version 5.01), ImageJ (National Institutes of Health, version 1.50i) and Excel (Microsoft, version 2016).

For manuscripts utilizing custom algorithms or software that are central to the research but not yet described in published literature, software must be made available to editors/reviewers. We strongly encourage code deposition in a community repository (e.g. GitHub). See the Nature Research guidelines for submitting code & software for further information.

Data

Policy information about availability of data

All manuscripts must include a data availability statement. This statement should provide the following information, where applicable:

- Accession codes, unique identifiers, or web links for publicly available datasets
- A list of figures that have associated raw data
- A description of any restrictions on data availability

Data available on request from the authors (Methods/Data Availability)

Field-specific reporting

Please select the one below that is the best fit for your research. If you are not sure, read the appropriate sections before making your selection.

☒ Life sciences ☐ Behavioural & social sciences ☐ Ecological, evolutionary & environmental sciences

Life sciences study design

All studies must disclose on these points even when the disclosure is negative.

Sample size	Sample size was chosen depending on the experimental approaches used based on our experience and estimation (Methods/Statistical Analysis). No power calculations were performed to choose group size (Methods/Mice).
Data exclusions	All animal samples were included in analysis (Methods/mice) except the experiments in Fig. 3P, given that some tumors in WT groups are almost invisible for dissection.
Replication	Most experiments were repeated at least two times and the representative result is presented. The number of biological replicates used in statistical analyses is stated in figure legends.
Randomization	For all mouse xenograft assays, the same cells were randomly injected into subcutaneous regions into randomly picked nude mice. No particular randomization method was used to assign animals to experimental groups (Methods/Mice).
Blinding	Investigators were not blinded during the experiment or when assessing the outcome (Methods/mice).

Reporting for specific materials, systems and methods

We require information from authors about some types of materials, experimental systems and methods used in many studies. Here, indicate whether each material, system or method listed is relevant to your study. If you are not sure if a list item applies to your research, read the appropriate section before selecting a response.

Materials & experimental systems

n/a	Involved in the study	
☐	☒	Antibodies
☐	☒	Eukaryotic cell lines
☒	☐	Palaeontology
☐	☒	Animals and other organisms
☒	☐	Human research participants
☒	☐	Clinical data

Methods

n/a	Involved in the study	
☒	☐	ChIP-seq
☒	☐	Flow cytometry
☒	☐	MRI-based neuroimaging

Antibodies

Antibodies used	Description of antibodies and other materials can be found in Methods/Materials section. Anti-SAV1 antibody (cat#13301, lot#1), anti-phospho-Thr308-Akt antibody (cat#2965, lot#18), anti-phospho-Ser473-Akt antibody (cat#4060, lot#19 and 23), anti-Akt1 antibody (cat#2938, lot#4; and cat#2967, lot#17), anti-phospho-Ser9-GSK3b antibody (cat#5558, lot#6), anti-PTEN antibody (cat#9559, lot#17), anti-phospho-Thr389-S6K antibody (cat#9234, lot#11), anti-S6K1 antibody (cat#2708), anti-GST antibody (cat#2625, lot#7), anti-phospho42/44-ERK (cat#4370, lot#17), anti-phospho-Thr180/Tyr182-p38 (cat#9211, lot#21), anti-Axl antibody (cat#8661s, lot#4), anti-Phospho-Y702-Axl (cat#5724s, lot#1), anti-Tyro3 antibody (cat#5585, lot#2) and polyclonal anti-phospho-Tyr1086-EGFR antibody (cat#2220s, lot#2) were purchased from Cell Signaling Technology. Polyclonal anti-HA antibody (cat#sc-805, lot#K1215) was obtained from Santa Cruz. Polyclonal anti-Flag antibody (cat#F-7425, lot#078M4886V), monoclonal anti-Flag antibody (cat#F-3165, clone M2, lot#SLBN8915V), monoclonal anti-Tubulin antibody (cat#T-5168, lot#115M4828V), monoclonal anti-Vinculin antibody (cat#V9131), anti-Flag agarose beads (cat#A-2220, lot#SLBW1929), anti-HA agarose beads (cat#A-2095, lot#057M4864V), peroxidase-conjugated anti-mouse secondary antibody (cat#A-4416, lot#SLBW4917) and peroxidase-conjugated anti-rabbit secondary antibody (cat#A-4914, lot#SLBV6850) were obtained from Sigma. Anti-MERTK and anti-EGFR antibodies were generated by the Earp lab. The polyclonal Akt1-pY26 antibody was generated by Abclonal, Inc derived from rabbits and validated.
Validation	All antibodies used in the study are commercially available and have been validated according to manufacturers manuals. Several antibodies were re-evaluated in the present study by immunoblot analysis including SAV1, MERTK, Alx, Tyro3, Akt1 and Akt1-pY26.

Eukaryotic cell lines

Policy information about cell lines

Cell line source(s)	HeLa cells were as described in Liu et al. 2014. Nature. 508: 541-548. HEK293T and other RCC cell lines were as described in Zhang et al. 2018. Science. 361: 290-295.
Authentication	Cell lines were not authenticated.

Mycoplasma contamination	HEK293T cells were tested to be mycoplasma free. None of the RCC cell lines used in the study were tested in this study but prior to this study.
Commonly misidentified lines (See ICLAC register)	No commonly misidentified lines were used in this study.

Animals and other organisms

Policy information about studies involving animals; ARRIVE guidelines recommended for reporting animal research

Laboratory animals	4-6 week old male and female nude mice were purchased from UNC Lineberger Animal Studies Core and maintained by UNC Animal Model Core Facilities. All experimental procedures are approved by UNC IACUC.
Wild animals	not available to this study.
Field-collected samples	not available to this study.
Ethics oversight	All of the necessary animal procedures were performed at the UNC mouse facility, located on the basement of the Genetic Medicine Building (GMB). The mice were housed and maintained under sterile conditions in facilities approved by the American Association for the Accreditation of Laboratory Animal Care and in accordance with current regulations and standards of the United States Department of Agriculture, United States Department of Health and Human Services, and the NIH. The mice were used in accordance with Animal Care and Use Guidelines of The University of North Carolina at Chapel Hill under protocols to Drs. Pengda Liu and William Y. Kim approved by the Institutional Animal Care and Use Committee.

Note that full information on the approval of the study protocol must also be provided in the manuscript.
